# *Plac1* Ablation Disrupts Signaling Pathways Essential for Prenatal Development and Induces a Preeclampsia-associated Transcriptomic Signature

**DOI:** 10.64898/2026.04.30.721637

**Published:** 2026-05-28

**Authors:** Suzanne Jackman, Xiaoyuan Kong, Yulan Piao, Alexei Sharov, Elin Lehrmann, Andrew Varshine, Ramaiah Nagaraja, David Schlessinger, Michael E. Fant

**Affiliations:** 1 Department of Pediatrics, University of South Florida, Morsani College of Medicine, Tampa, FL; 2 Laboratory of Genetics and Genomics, National Institute on Aging, National Institutes of Health, Baltimore, Maryland

**Keywords:** Plac1, placental development, Rho GTPase signaling, fetal growth restriction (FGR), birth defects, cardiovascular disease, preeclampsia, brain development, Developmental Origins of Health and Disease (DOHaD)

## Abstract

*Plac1* is an X-linked gene essential for placental and embryonic development. A knockout (KO) mouse model was used to identify *Plac1*-regulated gene expression at E16.5 and E18.5 using gene expression microarray. Genes exhibiting at least 1.5-fold change in expression and FDR < .05 were considered significant. At E16.5, 717 genes were downregulated and 798 were upregulated in male KO placentas versus wild type (WT), whereas at E18.5, 1122 genes were downregulated and 1149 were upregulated. GO, KEGG, and IPA analyses revealed downregulated genes were enriched for Rho GTPase-mediated and actin-cytoskeleton based processes that transmit extracellular cues through canonical signaling pathways, including Integrin, GPCR, Wnt, Notch, VEGF, BMP and TGF-beta, documented to impact trophoblast development, vasculogenesis, vascular tone, branching morphogenesis, and immunomodulation. Furthermore, a preeclampsia transcriptomic-associated signature was induced that strengthened over time. By contrast, upregulated genes reflected immune activation and adaptations to oxidative stress resulting from impaired placental function. These findings indicate that Plac1 supports signaling required to maintain placental structure and regulatory function. Its absence disrupts essential regulatory processes and triggers cellular stress and immune activation, contributing to fetal growth restriction, increased risk for embryopathy and preeclampsia, consistent with the Developmental Origins of Health and Disease (DOHaD) framework.

## 1. Introduction

*Plac1* is an X-linked gene that maps to a chromosomal locus previously shown to be important for placental and embryonic development [1]. Its expression is particularly high in the placenta, hence its name **Plac**enta-specific **1,** as the placenta was initially thought to be the only locus of its expression [1,2]. Subsequent studies, however, have demonstrated that it is also expressed throughout the developing embryo, albeit at 1–10% of placental expression levels, suggesting a broader role in embryonic development. Its expression in adult animals, however, diminishes to essentially undetectable levels [3]. The human PLAC1 gene encodes a putative protein of 212 amino acids, whereas the mouse ortholog has 173 amino acids with 65% homology [1]. The protein is predicted to exist as a membrane-associated or extracellular peptide (PSORT: psort.ims.u-tokyo.ac.jp). Consistent with targeting to a membranous compartment, a predicted transmembrane (TM) domain lies at aa 23 – 40 from the N-terminus. Within the placenta, *PLAC1* is expressed exclusively in the trophoblast, where its expression is tightly linked to differentiation of the syncytiotrophoblast [1,2,4]. Immunohistochemical analysis of cytotrophoblasts has demonstrated distribution throughout the cytosolic region as well as near the plasma membrane. By contrast, its localization in the differentiated syncytiotrophoblast is highly restricted to the apical cytosolic region in proximity to the maternal-facing microvillous membrane surface [5].

An essential role for *Plac1* in placental and embryonic development has been demonstrated in a mutant mouse model [6]. Placentas in *Plac1*-null mice exhibit placentomegaly with an enlarged and disorganized junctional zone (JZ), characterized by spongiotrophoblast (SpT) hyperplasia and associated with mild fetal growth restriction. Consistent with preferential paternal X chromosome inactivation in murine extraembryonic tissue [7,8], genetic analyses revealed that placentas derived from maternal (X^m-^X) heterozygotes (Hets) were phenotypically similar (but not identical) to knockout (KO) placentas whereas paternal (XX^p-^) Hets were phenotypically identical to wild type (WT). Further analysis suggested that the paternal *Plac1* allele is not completely inactivated, with approximately 10–15% residual activity remaining, providing some degree of functional activity in the X^m-^X Het not observed in the KO.

A functional role for Plac1 was also shown to extend to the embryo proper. Surviving *Plac1* mutants (*Plac1*-null males and X^m-^X females) developed postnatal hydrocephalus with increased frequency, 22% and 11% respectively, suggesting Plac1 plays an important role in fetal brain development. The changes in the placental phenotype were later supported by Muto, et al, using a mutant mouse model derived using a different targeting vector and bred against a different mouse strain [9]. By employing Lentivirus-mediated *Plac1* expression, they were also able to show that Plac1 rescue failed to reverse the overgrowth of the spongiotrophoblast layer but did ameliorate changes in the labyrinth, pointing to temporal aspect(s) of Plac1 function.

PLAC1 expression has also been shown to be reactivated in a wide range of human cancers and cell lines in which it influences cell proliferation and migration *in vitro.* Its *in vivo* expression in tumors has been linked to increased metastasis risk and decreased survival [reviewed in 10].

We applied our mutant mouse model to the identification of genes and signaling pathways regulated by Plac1 that drive placental development and embryogenesis. Given the high degree of conservation of Plac1 between mouse and human, and its highly restricted, trophoblast-specific expression, Plac1 is likely to occupy an upstream position within regulatory hierarchies that coordinate placental growth, differentiation, and maternal–fetal exchange. Accordingly, the objective of this study was not to resolve the full regulatory cascade downstream of Plac1, but rather to establish a framework describing the physiological and signaling landscapes perturbed by its loss. By integrating gene-level expression changes with pathway and systems-level analyses, we aimed to identify the core biological processes dependent on Plac1 during late gestation and to generate a cohesive, hypothesis-generating model of its role in placental development and pregnancy maintenance. Accordingly, this study combines gene-centric analysis of highly dysregulated transcripts and reciprocally regulated genes with complementary GO, KEGG, and IPA analyses. This integrative strategy was designed to facilitate interpretation by multidisciplinary groups of investigators and to establish a biologically coherent framework upon which future mechanistic studies can build.

## 2. Results

### 2.1 Developmental dynamics of placental growth in Plac1 mutants

**Figure 1.**
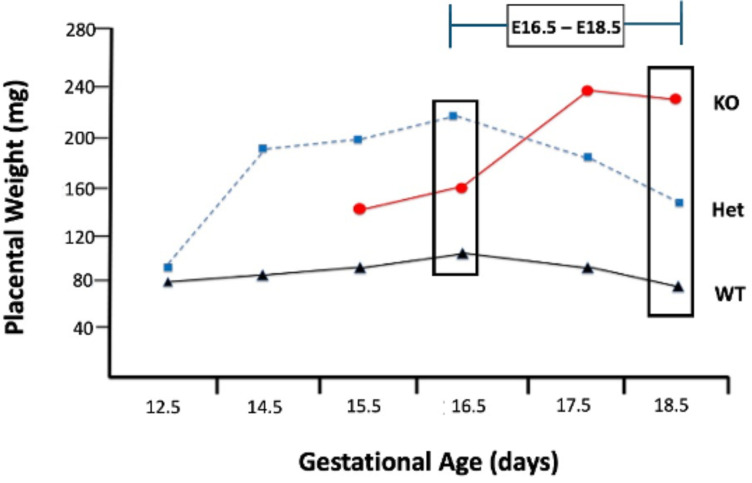
Growth trajectories of placentas from Plac-1 mutants compared to WT placentas. Placental weights were determined throughout gestation representing WT, X^m-^X, and KO mice and presented in graphical form summarizing previously published data [6]. Each point represents the mean of age-specific samples for each genotype (n = 1–10 per data point). WT and KO data include both male and female placentas.

We focused this analysis on the period of pregnancy when the growth trajectory of *Plac1-*null placentas exhibit maximal divergence from WT placentas. Placentas associated with *Plac1*-null embryos and maternal Hets (X^m-^X) exhibit placentomegaly and a disorganized junctional zone (JZ) [6]. Placental weights of X^m-^X Hets diverge from KO littermates between E16.5 and E18.5, likely because the paternal allele escapes complete inactivation. As we previously reported [6] and summarize in Figure 1, X^m-^X Het placental weight peaks at E16.5 and decreases slightly thereafter. By contrast, the weight of *Plac1-*null placentas continues to accelerate until E17.5 and then plateaus. These observations guided our transcriptomic analysis of the developmental span bordered by E16.5 and E18.5.

### 2.2 Gene Expression Microarray Analysis

The Agilent 4×44k gene chip was used to identify *Plac1-*dependent gene expression at E16.5 and E18.5. KO male placentas were compared to WT male placentas in duplicate samples. At E16.5, 717 known or putative genes were downregulated and 798 genes were upregulated at least 1.5-fold (FDR < .05) in KO placentas compared to WT. Similarly, at E18.5, 1122 genes were downregulated and 1149 genes were upregulated. (See supplemental materials, Tables S1-S7, for processed data and complete gene lists).

**Figure 2.**
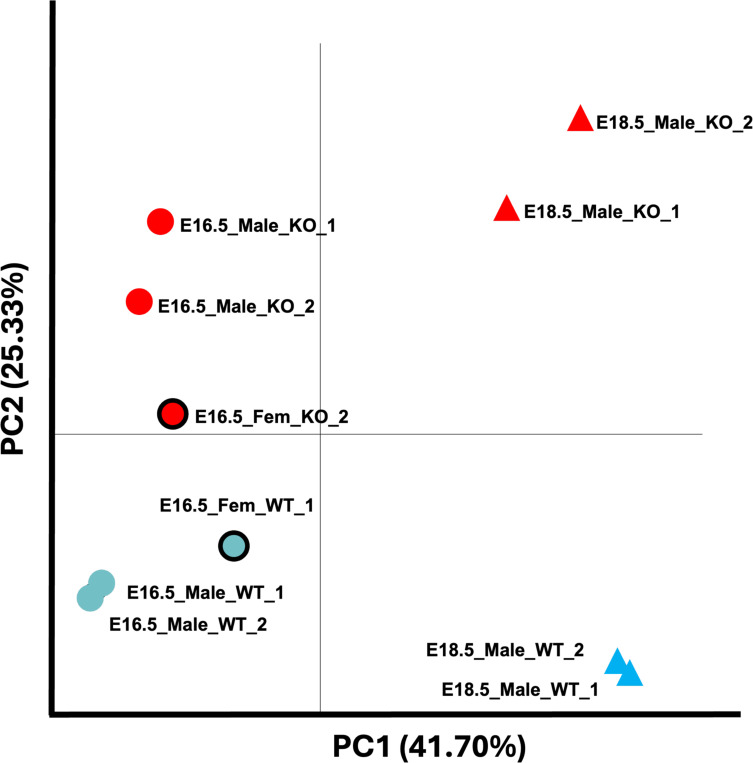
PCA of normalized expression data from E16.5 and E18.5 placentas (WT and KO). PC1, and PC2 explain [41.70%] and [25.33%] of the variance, respectively. PC1 separates E16.5 vs E18.5, PC2 separates WT vs KO. Points are labeled by age, sex, genotype, and replicate. Color = genotype (WT = blue, KO = red); Shape = age (E16.5 = circles, E18.5 = triangles); Black outline = female. Note there is only one female replicate per genotype and are shown for descriptive purposes only. No statistical or comparative conclusions were drawn due to the lack of biological replication.

Principal Component Analysis (PCA) (Supplemental Table S3) revealed that the first three components explained 79.1% of the variance (PC1: 41.7%, PC2: 25.33%, PC3:12.07%). Visual inspection of PC1 and PC2 (Figure 2) indicates that PC1 primarily separated samples by gestational age (E16.5 versus E18.5, whereas PC2 distinguished genotypes (WT from KO). Female samples are included to provide reassurance regarding the overall coherence and quality of the expression data derived from the male samples and are shown for qualitative visualization only. No conclusions were drawn due to the lack of biological replication. The WT female sample appeared modestly separated from the KO female, suggesting a potential female specific expression pattern (possibly contributing to PC3). Because *Plac-1* is X-linked, subtle sex dependent effects are not unexpected. However, this observation should be interpreted cautiously given the lack of female replicates.

**Figure 3.**
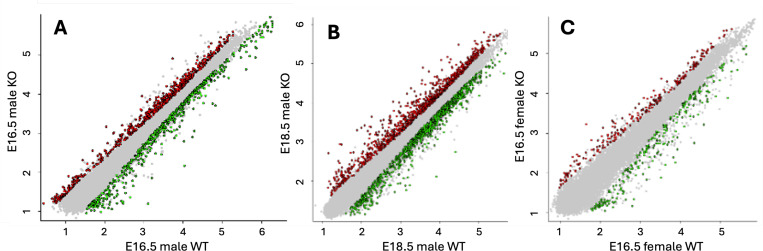
KO vs WT scatter plots by age and sex. Pairwise scatter plots compare KO (y-axis) versus WT (x-axis) log10 expression within **(A)** E16.5 males (replicate means), **(B)** E18.5 males (replicate means), and **(C)** E16.5 females. Single female samples are shown for descriptive purposes only. No statistical or comparative conclusions were drawn due to lack of biological replication. Each point represents a gene/probe; the diagonal line indicates y = x (equal expression in KO and WT). Red points represent upregulated genes in the KO; green points represent downregulated genes in the KO; grey points represent no significant dysregulation, based on ≥1.5-fold change and FDR < 0.05.

KO versus WT scatter plots are shown for each age/sex group (Figure 3). Most genes lie near the diagonal reference line, indicating broadly similar expression between KO and WT. Colored points denote differentially expressed genes (DEGs) meeting our threshold (≥1.5-fold change, FDR < 0.05). Red indicates upregulated genes in the *Plac1* KO and green indicates downregulated KO genes. Grey points indicate genes not significantly dysregulated. Axes show log_10_ expression values. Panel A and panel B display replicate means for males, whereas panel C shows values from one female per genotype.

**Figure 4.**
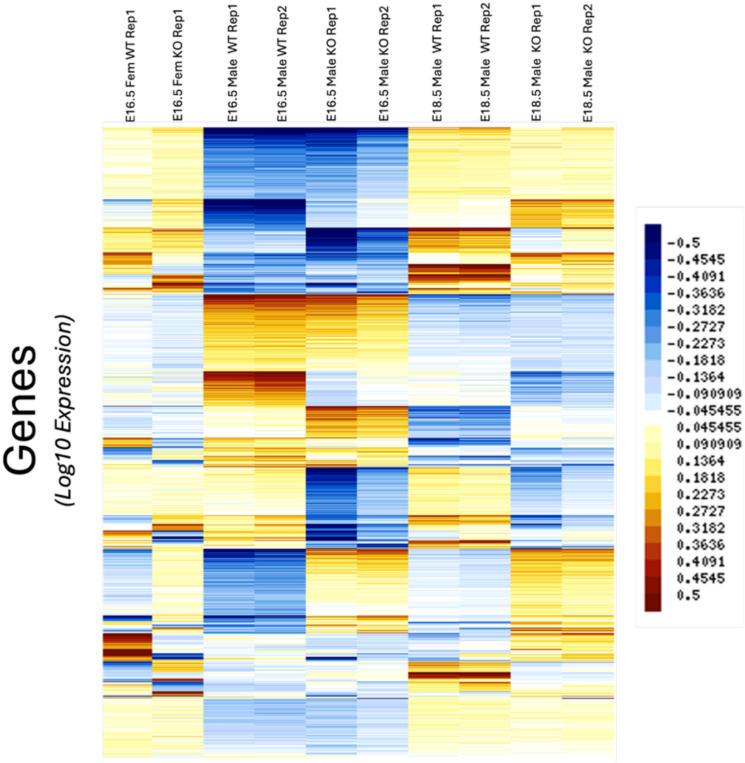
Heatmap of differentially expressed genes across samples. Heatmap displays relative gene expression patterns for DEGs identified at each developmental stage. For visualization, log_10_-transformed expression values were row-centered such that each gene’s mean expression across all samples equals zero. Color indicates expression relative to that gene’s mean level (blue, lower than average; yellow/brown, higher than average). Values were not variance-scaled. Genes (rows) are ordered by hierarchical clustering.

The heatmap of DEGs meeting our cutoff criteria of ≥1.5-fold change and FDR < 0.05 is shown in Figure 4. Differential expression was assessed using linear mixed-effects model with subjects treated as a random effect. Expression values are visualized, with genes (rows) ordered by hierarchical clustering. Although hierarchical clustering was applied only to genes and not to samples, several gene clusters exhibit expression patterns that appear to align with trends observed in the PCA. Specifically, a subset of genes revealed slightly divergent expression in WT versus KO females. However, again because of the lack of female replicates, a possible sex-associated trend should be interpreted cautiously.

### 2.3 RT-qPCR Confirmation of Directional Differential Expression

**Figure 5.**
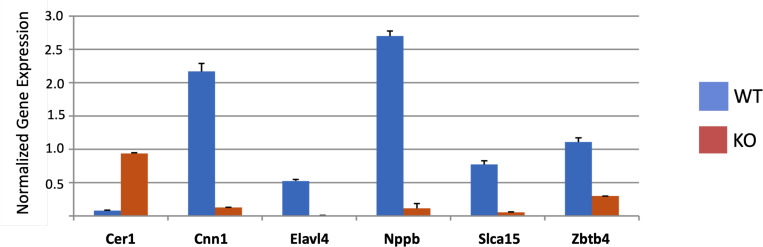
Real-time qRT-PCR of selected placental genes. Two biological replicates per genotype were assayed in triplicate. Bars represent the mean of two biological replicates per genotype (n = 2) ± SEM. Values for each gene were independently rescaled to permit visualization within a shared panel; absolute values cannot be compared between genes. *qRT-PCR results are shown as qualitative confirmation of expression direction. Statistical significance was not recalculated due to unavailability of original raw data. The original single-gene plots with native scaling are provided in Supplementary File S1.

A panel of genes was selected to validate the original microarray results when it was first carried out. Five downregulated genes (Cnn1, Elavl4, Nppb, Slc6a15, Zbtb4) and one upregulated gene (Cer1) were assessed in placentas at E18.5 (Figure 5). The results were qualitatively consistent in direction with the current (2026) microarray analysis. Accordingly, qRT-PCR results are presented as confirmation of the direction of differential expression rather than as independent statistical validation, as the original raw data are no longer available for re-analysis. These data represent the mean of two biological replicates assayed in triplicate per genotype (See Supplemental Figure S1).

Diminishing availability of mutant placentas due to colony attrition limited the number of genes that could be assayed. While these results are supportive of our microarray expression data, we acknowledge the limitations imposed by the small sample size on performing a robust statistical analysis. Surveying a larger sample of genes derived from multiple placentas will be necessary to establish a statistically powered qPCR validation in future studies.

### 2.4 Gene-Level Curation of Dysregulated Genes

Given the complexity of placental development and its sensitivity to temporal and physiological context, we initially approached the Plac1-regulated transcriptomic changes by manually curating a limited subset of highly dysregulated and developmentally informative genes. Importantly, these uniquely dysregulated subsets represent the unbiased identification of genes that may contribute to pathophysiological responses to Plac1’s absence. This gene-centric approach provides a time-resolved physiological perspective, highlighting directionality, adaptation, and potential compensatory programs that can otherwise be obscured when transcripts are immediately aggregated into higher-order categories. We then applied GO, KEGG, and IPA analyses to gain a systems-level interpretation, allowing pathway-level structure to be layered onto a biologically grounded narrative derived from the most salient gene-level changes.

DEGs exhibiting the highest fold-change at E16.5 and E18.5 were examined for potential relevance to the *Plac1*-null phenotype (See Supplemental Tables S4–S7 for complete gene lists). Their hierarchical order differed at each gestational age, reflecting the placenta’s intrinsic developmental program and its adaptations to deficits resulting from *Plac1’s* absence. We also identified small subsets of DEGs showing reciprocal directional dysregulation over time (e.g., downregulation at E16.5 followed by upregulation at E18.5) and examined these patterns as possible indicators of evolving pathophysiology. Functional inferences that emerged are intended to suggest plausible interpretations based on known or inferred gene function as well as placental physiology. Definitive interpretation will require experimental validation in the future.

#### 2.4.1 Downregulated Genes: Inferred Functional Deficits

To provide biological context for the most prominent transcriptional changes, we first focused on a small subset of the most strongly downregulated genes at E16.5 and E18.5. Many of these genes are linked to immune–trophoblast interactions, membrane or vesicular processes, endocrine signaling, and transcriptional programs associated with late placental maturation. Discussion of these genes individually offers insight into potential functional consequences of Plac1 loss at the level of specific cellular programs, prior to the broader pathway-based analyses.

At E16.5, KO placentas showed marked decreases in granzymes (***Gzmc***, ***Gzmf***, ***Gzmg***; and ***Gzmd*** ~13-fold) versus WT (Table 1). Granzymes are produced by uterine natural killer cells (uNKs), not trophoblasts. uNKs promote spiral artery remodeling by facilitating invasive trophoblast differentiation and depth (11–13). Their reduction therefore suggests either fewer uNKs or reduced uNK–trophoblast encounters needed to induce granzyme expression. Pertinent to this, PLAC1 has been shown to interact directly with NK activating proteins (NKG2D, NKp30, NKp44, DNAM-1) and can modulate NK cytotoxicity [14]. If reduced trophoblast invasion occurred early in placentation, consistent with PLAC1’s role in trophoblast invasion/migration in culture [15], uNK activation would be limited, impairing spiral artery remodeling and contributing to later placental insufficiency.

A second potential explanation is impaired recruitment of uNKs to the maternal–placental interface. **Cxcr4**, a GPCR for **Cxcl12**, mediates uNK homing to decidua, and Cxcr4 deficiency reduces uNK numbers, disrupts placental vasculature, and causes pregnancy loss [16]. Although neither gene is dysregulated in our study, the E16.5–E18.5 window occurs well after early placentation and would not be expected to capture earlier recruitment signatures. Interestingly, ***Tff3*** (second most downregulated) can also bind Cxcr4 and promote epithelial recruitment in retina [17], but there is no evidence it acts as a gestational chemoattractant. Instead, **Tff3** protein is expressed across multiple mouse epithelia at E14–E18, contributing to maturation and barrier formation [18]. However, there is no evidence of meaningful expression in placental cell populations based on STAMP analysis.

Evidence of early vascular impairment is further strengthened by disruption of the trophoblast–vascular interface. ***Angptl3*** downregulation suggests impaired nutrient delivery and labyrinthine function [21–23]. Its C-terminal domain binds integrin αvβ3 to promote endothelial adhesion/migration/vessel formation. Reduced expression would therefore limit migration and capillary branching. **Angptl3** also inhibits endothelial lipase and LPL, potentially affecting lipid availability and essential fatty-acid delivery to the fetus. In parallel, ***Ehd4***, an intracellular trafficking protein, participates in a PACSIN2/EHD4/MICALL1 complex controlling VE-cadherin recycling during sprouting angiogenesis [24]. Together, these changes point to the possible loss of regulatory integrity between trophoblasts and the endothelium.

Stress-related compromise was suggested by the profound downregulation of ***Krt6a***, typically induced in stratified epithelia in response to stress [25] and implicated in abnormal placentation (placenta accreta) [26]. Here ***Krt6a*** is ~45-fold reduced, consistent with decompensated stress, and is accompanied by reduction of additional keratins, ***Krt8*** and ***Krt18***, suggesting late-gestation trophoblast instability. Downregulation of ***Ceacam18***, expressed in decidua, spongiotrophoblasts, sTGCs, and labyrinthine trophoblasts, further suggests membrane disruption. While its placental function is unknown, other Ceacam family members support roles in adhesion and immunomodulation [27].

Genes affecting fetoplacental metabolism are also dysregulated. ***Dio2*** converts T4 to the active form, T3. T3 supports neuronal differentiation and trophoblast cell cycle progression, migration, and invasion. Early ***Dio2*** downregulation has been linked to shallow invasion and miscarriage [28]. Because fetal thyroid output is still increasing during this time, reduced placental ***Dio2*** at E18.5 implies reduced fetal T3 availability during a critical neurodevelopmental window. Placental ***Isx*** is also profoundly reduced, likely disrupting β-carotene–dependent regulation of *Mtp* and *ApoB* transcription [29], impairing lipoprotein-mediated provitamin A transfer. Since β-carotene supports *in situ* retinoic acid essential for organogenesis, impaired transfer may constrain retinoic acid during late gestation when fetal demand peaks, potentially compromising maturation of pulmonary, renal, and neural systems.

Notably, several downregulated genes (***Elavl4, Bhlhe22, Ntrk2, Neurod4, Vmn1r17***) are typically linked to neurological development. ***Elavl4*** is an early marker of neuronal differentiation and contributes to synaptic plasticity [30]. Its juxta-placental expression is confined to the parietal endoderm and was negligible in trophoblast populations (STAMP). Given its restricted expression to parietal endoderm, its reduction in KO placentas possibly reflects disruption of this specialized extraembryonic compartment, consistent with altered architecture and function of the placental boundary rather than trophoblast-intrinsic transcriptional effects. ***Ntrk2*** (TrkB), classically the high-affinity receptor for BDNF in nervous system development [31], is also expressed in trophoblasts along with its ligands throughout gestation [32]. Trk inhibition in pregnancy suppresses placental development, increases trophoblast apoptosis, reduces labyrinth area at mid-gestation, and decreases fetal weight later in gestation. ***Bhlhe22,*** a basic helix-loop-helix transcriptional regulator associated with neuronal differentiation and sensory specification (33,34) has no known placental function. It was identified among the most downregulated genes in our microarray analysis. However, bulk RNA-seq/Affymetrix (Bgee) and single-cell STAMP profiling showed no meaningful expression across placental cell populations. Similarly, ***Neurod4***, a neuronal differentiation factor [35], and ***Vmn1r17***, a pheromone-related receptor (36), lack evidence for placental expression. Given that *Plac1* is expressed in both fetal brain and placenta and the KO is not placenta-specific, its apparent dysregulation (and that of *Neurod4 and Bhlhe22*) could reflect contributions from non-placental tissues, including brain-derived transcripts or extracellular vesicles (EVCs) entering the placental circulation [37]. However, mapping artifacts or low-level, non-specific microarray signal remain plausible alternative explanations and require independent validation. Finally, ***Or51e2*** (mouse ortholog ***Olfr78***) is expressed in smooth muscle cells of resistance vessels, sensing small-chain fatty acids (SCFA) and metabolites to regulate vascular tone [38] and renin secretion [39]. It localizes to mesenchymal and decidual stromal cells (STAMP) where it may play a role in paracrine regulatory interactions.

Taken together, the coordinated downregulation of these genes suggests that Plac1 deficiency disrupts multiple late-gestational programs required for placental maturation, including immune signaling, membrane trafficking, and endocrine linked regulatory processes. Rather than reflecting isolated gene-level effects, these changes likely point to a broader impairment in the functional specialization of the placenta that may help contextualize pathway-level signatures.

#### 2.4.2 Upregulated Genes: Adaptations and Stress Responses to Functional Deficits

In contrast to the genes suppressed in the *Plac1*-null placenta, the most strongly upregulated genes at E16.5 and E18.5 suggest activation of stress-responsive, immune-associated and compensatory developmental programs. These genes span transcriptional regulators, immune receptors, metabolic enzymes, and poorly characterized loci, consistent with adaptive responses to declining placental functional reserve. Consideration of these genes individually provides insight into how the placenta may be attempting to stabilize fetal support as structural and signaling integrity becomes compromised.

At E16.5, the KO placenta shows strong induction of oxidative stress and immune activation genes, including robust ***Igfbp1*** upregulation, consistent with reduced oxygen/nutrient availability and compensatory IGF signaling [40]. Increased ***Ifi203*** suggests activation of type I interferon pathways under cellular stress [41]. Upregulation of ***Tcra*** may reflect maternal immune cell infiltration as inflammatory pressure increases [42]. Markers of lineage instability also emerge, notably ectopic expression of ***Khdc1b***, an oocyte/early embryo KH-domain protein that regulates maternally stored mRNA stability/translation [43]. Its induction in the placenta is consistent with stress-associated trophoblast dysfunction.

Induction of neuronal modules (***Syt15, Gabrq, Farp1***) may reflect derepression of noncanonical placental programs in a stressed environment. ***Syt15*** (synaptotagmin family) likely supports calcium-regulated membrane trafficking/exocytosis needed under increased transport demands (UnitPro: Syt15). ***Gabrq*** upregulation may mirror aberrant activation observed in cancer under hypoxic/metabolic stress [44]. ***Farp1***, a cytoskeletal membrane linker and Rho GEF (Rac1 activator), promotes dendritic growth and motility [45]. Its induction may represent an attempt to reinforce labyrinth structure and vascular architecture. ***Minar1*** (Membrane Integral NOTCH2 Associated Receptor 1) may represent a compensatory mechanism to dampen growth/angiogenesis/metabolic load in a resource-limited environment by suppressing mTOR and inhibiting angiogenesis while stabilizing Notch2 [46].

Finally, the lncRNA ***C730014E05Rik*** lies ~17.3 kb downstream of ***Adipoq***, placing it within an adiponectin-associated regulatory region (MGI:106675; MGI:2443371) [47,48]. Its stress-induced upregulation may influence transcriptional responsiveness within this domain [49]. Adiponectin is anti-diabetic/anti-inflammatory/anti-atherogenic and activates AMPK and downstream p38 MAPK signaling [50–53]. Because the placenta expresses adiponectin and its receptors, **AdipoR1/R2** [54,55], these pathways are relevant to trophoblast physiology. Consistent with this, multiple genes linked to AMPK, p38 MAPK, and TGFβ signaling (***Tgfb3, Eif4ebp1, Eef2, Pfkl, Pfkm, Mapk11, Mapk13***) were upregulated, raising the possibility that ***C730014E05Rik*** marks stress-related activation within the adiponectin locus aligned with AMPK/p38 and TGF-β induction.

By E18.5, disrupted trophoblast differentiation cues are suggested by elevated ***Ascl2***, essential for SpT development and GlyT expansion [56,57], along with induction of ***Cer1***, a Nodal/BMP/Wnt antagonist influencing trophoblast lineage allocation [58,59]. ***Ascl2*** upregulation aligns with the enlarged junctional zone in *Plac1*-null placentas and may reflect compensatory adjustment to altered lineage balance. Concordant upregulation of ***Spata21***, reported in placental Glycogen Cells (GC), GC precursors and JZ precursors (STAMP), as well as the placental labyrinth (Bgee), also aligns with expansion of JZ-associated populations. ***Cer1*** induction at E18.5 further supports continued SpT expansion.

Metabolic rewiring and lipid-mediated inflammation are suggested by increased ***Aldh1a3*** and ***Pla2g4d***. ***Aldh1a3*** is consistent with expanded GlyT populations [60,61], contributes to RA production, and detoxifies aldehydes generated during lipid peroxidation. Increased ***Pla2g4d*** (OMIM 612864) reflects ongoing lipid-mediated inflammatory stress. Heightened innate immune signaling is suggested by the induction of C-type lectins ***Clec2m*** and ***Clec9a***, and immunoproteasome subunit ***Psmb9***, reflecting interferon-mediated antigen processing and tissue injury. Because Clec9a senses F-actin from damaged cells and Psmb9 supports degradation of misfolded proteins, their induction is consistent with active cellular injury responses. Tissue-level stress is further supported by increased ***Lrfn2***, a synaptic adhesion molecule that may promote structural stabilization. Finally, ***1700027H10Rik***, a chromosome 3 lncRNA (MGI:1919524) with unknown function, was also induced.

Taken together, the genes most strongly upregulated at E16.5 and E18.5 indicate emergence of immune-associated, stress-responsive, and alternative developmental transcriptional programs that differ from those associated with normal placental maturation, reflecting a progressively altered placental gene expression landscape as gestation advances.

#### 2.4.3 Reciprocal Gene Dysregulation Over Time

A distinct gene subset exhibited reciprocal, gestational age-dependent dysregulation, being suppressed at E16.5 but induced by E18.5. These genes cluster into several functional categories related to protein homeostasis, endocrine and secretory activity, cytoskeletal organization, membrane architecture, and genome regulation. Examination of these reciprocal patterns provides insight into alterations in the temporal coordination of placental functional programs in the absence of Plac1, complementing the gene sets dysregulated at a single point in time.

Gestational age-dependent, reciprocally dysregulated genes reveal several functional modules in the KO placenta. First, as shown in Table 2A, downregulation of ***Dnajb11*** and ***Dnajc3*** at E16.5 suggests reduced unfolded-protein response (UPR) capacity during a critical growth window, whereas their coordinated upregulation at E18.5 likely reflects adaptive activation of proteostasis [62]. Second, the downregulation of placenta-specific prolactins (***Prl2c3, Prl7a2, Prl8a8***) and ***Rab27a*** implies diminished endocrine/secretory output followed by an attempt to restore hormonal signaling and exocytosis [63–66]. Notably, increased expression at E18.5 coincides with continued JZ expansion, where these genes are primarily expressed. Third, reciprocal regulation of ***Tmsb10*** (actin filament organization) and ***Prom1,*** an epithelial stem cell marker organizing apical micro-domains (including microvilli), may reflect a late attempt to reinforce trophoblast integrity and microvillous architecture.

***Noct*** regulates circadian metabolic control, NADP(H) phosphatase activity, and post-transcriptional control of metabolic mRNAs [67]. Although expressed in placenta (Uniprot: 035710; STAMP) without a defined placental role, its directional shift could reflect reduced metabolic regulation followed by an attempted compensatory response. Finally, ***Asz1*** is a germ cell-specific regulator essential for piRNA biogenesis, transposable element silencing, and proper meiotic progression (Uniprot: Q8VD46) [68,69]. Similarly, ***Gm773***, an X-linked gene, contributes to spermatogenesis and is required for sperm penetration of the zona pellucida [70]. Both are typically germline-restricted, yet show coordinated downregulation at E16.5 and upregulation at E18.5. This pattern may reflect progressive cellular stress followed by compensatory activation of genome-protection programs in response to worsening placental stress.

Collectively, the reciprocal expression patterns observed for this gene set suggest disrupted temporal regulation of multiple placental support programs rather than a uniform gain or loss of function. Early suppression followed by late induction of genes involved in proteostasis, endocrine signaling, vesicular trafficking, and cytoskeletal and membrane organization is consistent with perturbed engagement of late-gestational placental functions. Together, these data underscore altered temporal dynamics of placental gene regulation in the *Plac1*-null context, rather than simply magnitude-based changes in expression.

A second subset of genes exhibited the opposite reciprocal pattern, being upregulated at E16.5 but downregulated by E18.5. This group includes factors associated with extracellular matrix regulation, immune modulation, redox balance, and signaling pathways that act at the maternal–fetal interface. Analysis of this subset, shown in Table 2B, provides insight into placental programs that are engaged early but not sustained as gestation progresses in the absence of Plac1.

At E16.5, upregulation of ***Cnmd*** (anti-angiogenic ECM protein) [71,72] together with ***Spon1*** (matrix/adhesion cues relevant to cell attachment/axon guidance) [73] and ***Tfpi2*** (protease inhibitor suppressing trophoblast invasion) [74,75] can be viewed as attempts to stabilize the maternal–fetal interface. Concomitant induction of immune modulatory/tolerance factors, ***Crispld2*** (LPS-binding neutralizer), ***Gpnmb*** (inflammation-resolving glycoprotein) and ***Siglecg*** (inhibitory Siglec) suggests efforts to defend against inflammatory stress [76–79]. Upregulation of ***Gpx3***, ***H6pd***, and ***Sphk1*** points to adaptations to oxidative and ER stress [80–84]. Finally, induction of growth/genome maintenance genes, ***Porcn*** (Wnt ligand activity), ***Trnp1*** (progenitor proliferation) and ***Tatdn2*** (R-loop–resolving nuclease), is consistent with attempts to preserve trophoblast remodeling while containing replication stress [85–90]. Their downregulation by E18.5 possibly reflects exhaustion of these adaptive responses in a failing placenta.

Taken together, this reciprocal expression pattern suggests early engagement of multiple stabilizing and protective transcriptional programs that diminish by late gestation in the *Plac1*-null placenta. The coordinated downregulation of genes involved in extracellular matrix restraint, immune modulation, oxidative stress handling, and growth control at E18.5 is consistent with loss or attenuation of these early responses over time.

#### 2.4.4 Functional Classes of DEGs

Examination of the DEGs also revealed a marked enrichment for membrane-associated receptors, solute transporters, and ion channels pointing to broad disruption of membrane-linked signaling and transport functions in the *Plac1*-null placenta. (Table 3). Notably, many of these genes are also annotated to brain development, consistent with shared placental and neurodevelopmental regulatory programs and with the CNS abnormalities observed in *Plac1* knockout mice [3].

#### 2.4.5 Systems-level and Pathway Analyses: GO (Gene Ontology), KEGG (Kyoto Encyclopedia of Genes and Genomes), Ingenuity Pathway Analysis (IPA)

While examination of individual genes provides insight into specific temporal and functional perturbations, a systems-level perspective is required to more fully understand how these changes may align with broader biological processes. To this end, we next assessed global patterns of differential expression to identify shared functional themes and regulatory networks disrupted in the *Plac1*-null placenta using GO, KEGG, and IPA tools.

##### GO Analysis

**Figure 6.**
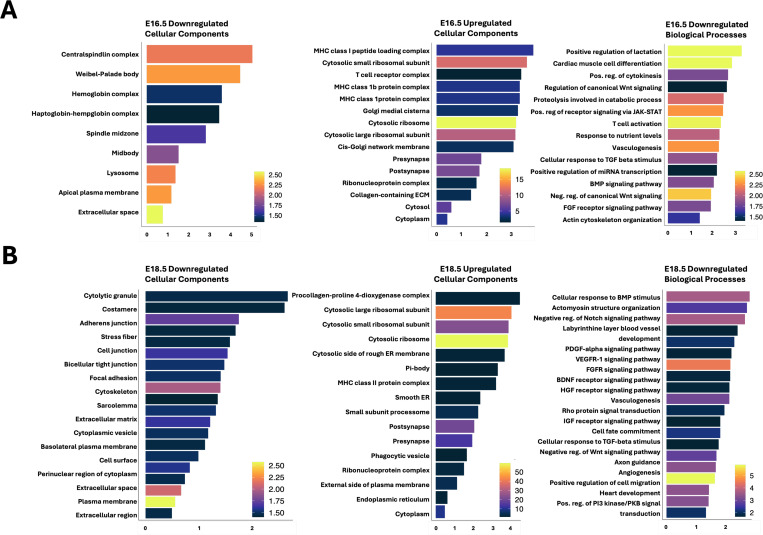
Developmental Stage-Specific Dysregulation of Cellular Components and Biological Processes in KO Placentas. Bars represent GO terms significantly enriched among the downregulated genes. The X-axis shows log_₂_(Enrichment), indicating fold enrichment relative to WT controls. The Y-axis lists individual GO terms. Bar colors correspond to –log_10_(FDR), with warmer colors (yellow/orange) indicating higher statistical significance and cooler colors (blue/purple/black) indicating lower significance. **Panel A:** E16.5, showing downregulated (left) and upregulated (middle) Cellular Components, and downregulated Biological Processes (right). **Panel B:** E18.5, showing downregulated (left) and upregulated (middle) Cellular Components, and downregulated Biological Processes (right). * Note – For visualization purposes, the number of BPs and CCs were selectively limited to a maximum of 20 by reducing implied functional redundancy.

GO enrichment demonstrated predominant downregulation of cellular components associated with the **membrane–cytoskeletal interface**, including plasma membrane, sarcolemma, lysosome, extracellular matrix, adherens and tight junctions, and actin-based structures at both E16.5 and E18.5 (Figure 6A, B; See Supplementary Tables S8–S13 for complete lists of GO Terms). These findings are consistent with Plac1 localization to membranous compartments, particularly at the apical trophoblast surface. Enrichment of midbody and spindle-associated structures suggested impaired cytokinesis, while prominent involvement of Weibel–Palade bodies at E16.5 indicated endothelial-specific dysfunction. Given their role in regulating the release of von Willebrand factor, angiopoietin-2, and endothelin-1, reduced WP-associated signaling would be expected to compromise hemostasis, vascular tone, angiogenesis, and immune regulation at the maternal–fetal interface [91–93].

In contrast, upregulated cellular components reflected stress-adaptive and compensatory responses, most notably enrichment of translational machinery, ribonucleoprotein complexes, and ER/Golgi compartments, particularly at E18.5. These changes indicate increased translational load, secretory activity, and proteostasis demand under nutrient and oxidative stress. Immune-related components also showed progressive enrichment, shifting from MHC class I and cytotoxic signaling at E16.5 toward MHC class II–associated antigen presentation, phagocytic vesicles, and adaptive immune activation by E18.5. Additional enrichment of extracellular matrix and synapse-annotated compartments, likely reflecting vesicle trafficking and cytoskeletal remodeling rather than neuronal specificity, further supported widespread disruption of membrane dynamics and cell–cell communication.

Downregulated GO biological process analysis revealed significant suppression of growth factor and developmental signaling pathways, including BMP, FGF, Wnt, TGF-β, and Jak–STAT, all critical regulators of placental development. In addition, genes dysregulated in our placental dataset were significantly enriched for GO annotations related to embryonic organogenesis. Cardiovascular, neurodevelopmental, pulmonary, renal, and skeletal pathways were disproportionately affected, consistent with *Plac1* expression in both the placenta and embryo and with documented neurodevelopmental vulnerability in *Plac1*-deficient models. GO Biological Process enrichment for upregulated genes at both E16.5 and E18.5 was represented by generalized immune, stress, and metabolic response terms that overlapped extensively with KEGG and IPA pathway annotations and is presented in full in the Supplementary Data (Tables S9, S12).

##### KEGG Analysis

**Figure 7.**
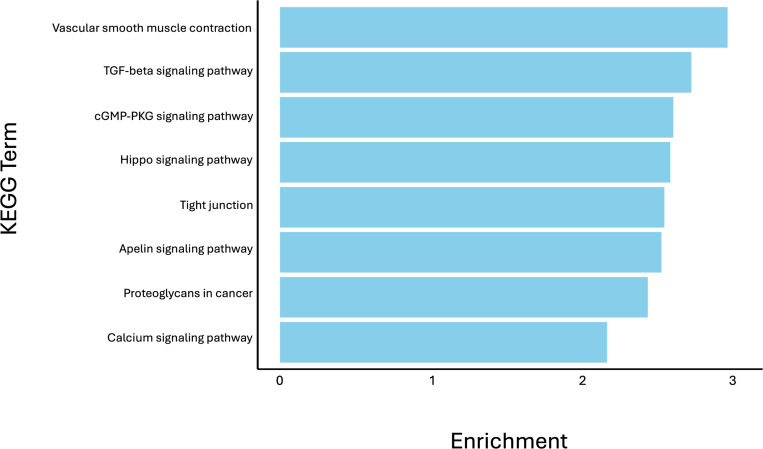
Fold enrichment of KEGG terms represented by downregulated genes at E18.5. Bars represent KEGG pathways significantly enriched among downregulated genes at E18.5. The X-axis shows fold enrichment, and the Y-axis lists individual KEGG terms (FDR < .05).

KEGG analysis at E18.5 reinforced these findings, identifying coordinated downregulation of pathways governing vascular smooth muscle contraction, calcium and cGMP–PKG signaling, apelin signaling, tight junction integrity, TGF-β signaling, and Hippo pathway components at E18.5 (Figure 7; See Supplementary Tables, S14–S18, for complete list of KEGG terms), collectively indicating impaired vascular remodeling, cytoskeletal organization, trophoblast proliferation, and structural stability. No significant downregulated KEGG terms were identified at E16.5.

Conversely, KEGG terms enriched among upregulated genes were dominated by cellular stress, metabolic adaptation, and immune activation, including **Ribosome**, **Protein Processing in the Endoplasmic Reticulum, Glycolysis/Gluconeogenesis, Carbon Metabolism**, **Phagosome**, and **Antigen Processing and Presentation** (Table 4, Supplemental Tables S14 and S17). Viral and inflammatory pathway annotations reflected heightened innate and adaptive immune signaling rather than pathogen-specific responses.

##### Ingenuity Pathway Analysis (IPA)

Broadening our systems-level interpretation of the *Plac1*-null transcriptome, we next applied IPA analysis. Unlike gene-level fold-change comparisons, IPA integrates both the directionality of gene expression changes and curated regulatory relationships among pathway components to predict pathway activity states. Canonical pathways were therefore evaluated using the IPA activation Z-score, which estimates whether the collective expression pattern of pathway-associated genes is more consistent with activation or inhibition relative to random expectation. This approach enables functional inference at the pathway level and complements the enrichment-based GO and KEGG analyses described previously.

**Figure 8.**
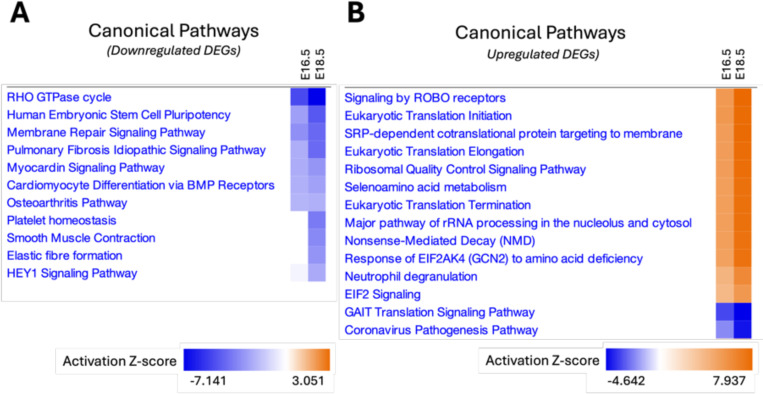
Developmental Stage-Specific Dysregulation of Canonical Pathways in KO Placentas **Panel A.** Canonical Pathways associated with **Downregulated** genes at E16.5 and E18.5. **Panel B.** Canonical Pathways associated with **Upregulated** genes at E16.5 and E 18.5. Color intensity reflects activation Z-scores: blue indicates negative Z-scores (predicted inhibition), and orange indicates positive Z-scores (predicted activation). All pathways shown have absolute Z-scores > 2 and Benjamini–Hochberg adjusted p-values < 0.05.

IPA “*Comparison Analysis”* (CA) identified **Rho GTPase signaling** as the most significantly downregulated pathway across both developmental stages (Figure 8A; see Supplementary Tables S19–S22 for complete pathway lists), along with myocardin signaling, smooth muscle differentiation, membrane repair, extracellular matrix remodeling, and embryonic stem cell pluripotency pathways [94–98]. Upregulated IPA pathways were highlighted by compensatory activation of translational control, ribosomal quality control, nonsense-mediated decay, EIF2/GCN2-mediated integrated stress response and antioxidant metabolism, consistent with chronic nutrient and oxidative stress [99–104] (Figure 8B; see Supplemental Table S23 and Figure S2). Suppression of GAIT and coronavirus pathogenesis pathways further suggested loss of translational restraint on inflammatory signaling, contributing to chronic placental inflammation and impaired immune tolerance [105,106]. Collectively, GO, KEGG, and IPA analyses converge on a model in which loss of *Plac1* disrupts membrane-proximal, actin-cytoskeletal, and vascular regulatory networks, triggering progressive placental stress, immune dysregulation, and functional decline.

#### 2.4.6 Downregulation of Developmental Signaling Annotated to Brain and Cardiovascular Systems

Numerous canonical pathways disrupted in the *Plac1* KO have been annotated, specifically, to the development and/or function of a variety of embryonic organs including lung, kidney, gastrointestinal system, musculoskeletal system and orofacial development. (See Supplemental Tables for downregulated IPA summaries: “Diseases and Biological Functions”, S24 and S25). These annotations reflect conserved developmental signaling pathways that are reused across multiple biological contexts, including placental development.

Pathways relevant to the structural and functional development of the cardiovascular system were disproportionately represented and strengthened over the course of gestation as indicated by their activation Z-scores and p-values (Table 5). We interpret these enrichments as reflecting disruption of shared regulatory signaling networks rather than direct evidence of cardiovascular development within placental tissue. Although no specific cardiovascular disorders were identified in our model, embryos and surviving mice were not systematically examined for defects that may have been present but otherwise not apparent. Notably, mice carrying the *Plac1*-null genotype carry an increased risk of fetal or perinatal lethality that increased to 100% lethality after multiple generations that may reflect disruption of signaling pathways with roles in multiple developmental systems, including cardiovascular development. While female Hets exhibited no increased lethality during early breeding they ultimately succumbed to progressive lethality as well.

The identification of pathways annotated to brain development was also notable in this context of shared developmental signaling, and in light of the increased risk of developing hydrocephalus in adult *Plac1* KO and Het mice. The L1cam Signaling Pathway is particularly interesting in this context. Mutations in L1CAM are the most common cause of X-linked hydrocephalus in humans and has been recapitulated in mouse models [107, 108]. Similar to *Plac1* mutants, the penetrance of hydrocephalus in *L1cam* mutants is variable suggesting multifactorial or polygenetic factors. *L1cam* localizes to the Xq28 genetic locus whereas *Plac1* localizes to Xq26.3, placing them in genomic proximity. Given both genes are independently associated with hydrocephalus, common or convergent developmental processes may be involved, although a direct mechanistic relationship cannot be inferred from the present data. Future studies of the *Plac1*-null model should systematically assess organogenesis in the developing embryos with particular emphasis on the cardiovascular and neurological systems.

#### 2.4.7 Induction of a Preeclampsia-associated Transcriptomic Signature

**Figure 9.**
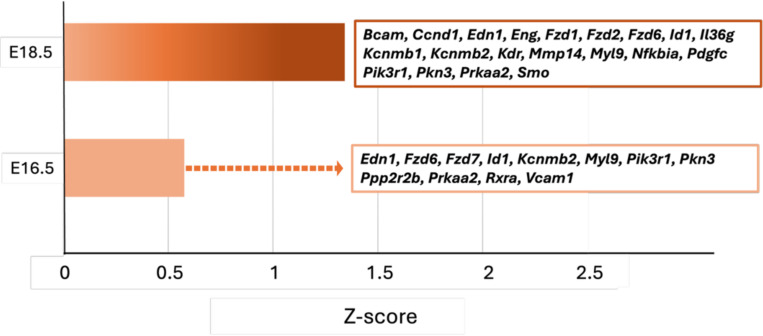
Preeclampsia Signaling Pathways Associated with Downregulated Genes at E16.5 and E18.5 in *Plac1*-null Placentas. Canonical pathways at each developmental age were identified using DEGs exhibiting at least a 1.5-fold decrease in expression compared to WT placentas. **E16.5** - Solid light orange color. (Z-score = 0.58, FDR = 0.19) **E18.5** - Dynamic orange fill depicting strengthening activation and significance level over time. (Z-score = 1.34, FDR = .03) **Text boxes:** DEGs populating the canonical PE pathway at each developmental age. The vertical axis denotes gestational age. The horizontal axis denotes positive Z-scores. The dashed line (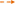) represents the dynamic shift toward activation from E16.5 to E18.5

The physiological relevance of *Plac1* during pregnancy was strengthened by the emergence of a statistically significant preeclampsia-associated (PE) signature at E16.5 that persisted and strengthened through E18.5 (Figure 9). Although the Z-score did not exceed the threshold to definitively predict activation (≥2.0) it was a statistically significant association and exhibited a clear dynamic shift in the positive direction (toward activation) during that time interval.

Additionally, a separate subset of DEGs identified at E16.5 and E18.5 (upregulated genes) were consistent with a clinically relevant biomarker for predicting the onset of PE during human pregnancy. Elevated maternal serum levels of glycosylated fibronectin, (GlyFn), have been validated as a highly predictive marker, predicting the onset of PE later in pregnancy (109). As shown in Table 6, placental fibronectin (*Fn1*) gene expression is significantly upregulated at E16.5 along with genes essential for post-translational glycosylation. Upregulation of *Fn1* together with *Galnt* family members, *B4galnt2*, *St3Gal3*, *St6gal1*, and *Mgat4a,* in the context of a placenta experiencing oxidative stress and inflammation, would be expected to increase the synthesis, O-glycosylation, sialylation, and secretion of cellular fibronectin, thereby increasing maternal serum glycosylated fibronectin and consistent with the mechanism underlying GlyFn elevation observed in preeclampsia. In fact, the appearance of *St6gal1* at E18.5 is notable because GlyFn assays use Sambucus nigra lectin which recognizes α2,6-sialylated epitopes, the moiety added by *St6Gal1* (110).

Unsupervised disease–gene enrichment analyses performed on our E18.5 downregulated dataset provided additional context for this pathway-level observation. DisGeNET analysis identified vascular diseases, hypertensive diseases, and vascular inflammation as significantly enriched disease classes (q = 0.02286 – 0.04728), with endothelial dysfunction showing borderline enrichment (q=.07406). By contrast, preeclampsia was only identified in the expanded OMIM library but did not reach the significance threshold after multiple-testing correction (p = 0.043, q = 0.077, OR = 2.05). Viewed collectively, these results support the convergence of shared cardiovascular and placental stress mechanisms that are associated with an elevated risk profile for PE rather than a categorical PE disease state. This interpretation is consistent with the IPA analysis, which indicates robust enrichment of vascular and endothelial stress programs central to preeclampsia pathophysiology, while falling just short of a disease-defining transcriptional signature during the time-frame studied.

When these studies were carried out the potential emergence of preeclampsia was not considered or assessed in the pregnant females. Therefore, the relevance of these observations to the clinical phenotype will need to be assessed in future studies. However, the temporal evolution of the Plac1-associated preeclampsia-like and vascular profiles, coinciding with an O-linked glycosylation signature that aligns with elevated serum levels of the PE biomarker, GlyFn, is consistent with its clinical manifestation in human pregnancy. This suggests that the developmental processes disrupted by the absence of Plac1 may be physiologically relevant to the pathogenesis of the human disease.

#### 2.4.8 Integrated Upstream Regulator Analysis Reveals Reciprocal Stress/Growth Rewiring

To further resolve the regulatory architecture underlying the *Plac1*-null transcriptome, we integrated upstream regulator profiles derived from IPA Comparison Analysis of upregulated and downregulated gene sets at E16.5 and E18.5. Selected results are shown in Table 7, reported as activation Z-scores and filtered for a B-H adjusted p-value < 0.05.

The downregulated dataset revealed coordinated inhibition of angiogenic, growth factor, and cytoskeletal regulators, including Vegf, Hgf, Igf1, and Tgfb1, as well as broader signaling nodes such as Fgf2 and Bmp4, together with Srf/Mrtfb, Yap1, and Tead2 family members, consistent with reduced vascular and developmental signaling. In contrast, the upregulated dataset was dominated by activation of inflammatory and stress-associated regulators, including Tnf, Il1b, Ifng, Rela, and Hif1a, alongside suppression of key anabolic and structural nodes such as Rictor, Larp1, and Lats. Notably, immune-associated regulators such as Tnf and Ifng were represented in both upregulated and downregulated datasets with opposing predicted activation states, indicating that subsets of their downstream targets are differentially induced or suppressed. This pattern is consistent with altered integration of cytokine-associated signaling pathways rather than uniform inflammatory activation. Together, these findings support a model of reciprocal regulatory shifts in which reduced vascular and developmental signaling is accompanied by selective activation of stress-responsive and inflammatory pathways. Complete upstream regulator results for both upregulated and downregulated gene sets are provided in Supplementary Tables S26–S29.

### 2.5 Electron Microscopy (EM) of Plac1-null Placentas

**Figure 10.**
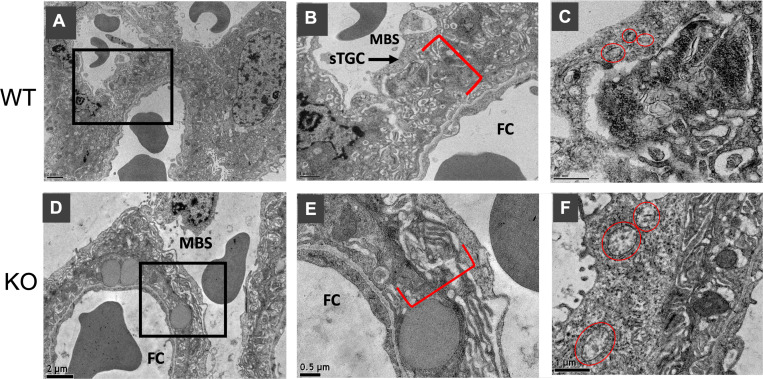
Transmission electron micrographs of the trilaminar interhemal region of E18.5 WT and KO placentas. **WT Panels:** The inset in panel **10A** shown at higher magnification in panel **10B**, depicts the trilaminar interhemal barrier separating the fetal capillaries (FC) from the maternal blood space (MBS). Sinusoidal trophoblast giant cells (sTGC; black arrow) are in direct contact with the MBS. A compact, well-defined region containing the syncytiotrophoblast (SynT) layers is indicated by the red brackets. Panel **10C** shows numerous small, well-defined mitochondria within an sTGC (red circles). **KO Panels:** Panels **10D** and **10E** show a similar interhemal region in the KO placenta. The SynT region appears more disordered and less compact. Panel **10F** depicts several enlarged mitochondria with poorly defined cristae (red circles). Scale bars = 0.5–2.0 μm.

E18.5 *Plac1*-null and WT placentas (one per genotype) were examined by electron microscopy. In the WT placenta (Fig. 10A–C), the labyrinth interhemal region displays a well-organized trilaminar architecture. Sinusoidal trophoblast giant cells (sTGCs) line the maternal blood space (MBS) (Fig. 10B), and the syncytiotrophoblast (SynT) region, indicated by the red bracket, forms an ordered layer overlying the basement membrane, followed by endothelial cells lining the fetal capillaries (FC). At higher magnification (Fig. 10C), mitochondria (indicated by red circles) are numerous and small (0.1–0.4 um) with discernible cristae.

By contrast, in the *Plac1*-null placenta (Fig. 10D–F), the corresponding region appears less compact and organized compared to the WT (red bracket, Fig. 10E), consistent with membrane-related perturbations suggested by the bioinformatic analyses. In addition, enlarged mitochondria (0.5−1.0 um) with poorly defined cristae are evident in KO sTGCs (Fig. 10F). This mitochondrial morphology was not observed in the WT fields examined, in which the mitochondria appear smaller and more uniform (Fig. 10C). Examination of additional *Plac1* mutant placentas will be necessary to validate and extend these findings. Source EM images are provided in Supplemental Figures S3–S8.

## 3. Discussion

The data presented suggest that Plac1 modulates membrane-associated signaling nodes coupling extracellular cues to cytoskeletal organization at the maternal–fetal interface. The loss of Plac1 induces significant placental stress and a progressive PE-like transcriptomic trajectory. Collectively, these findings position Plac1 as an upstream modulator of membrane-proximal regulatory systems integrating cytoskeletal dynamics, trophoblast function, vascular remodeling, and immune regulation during late gestation. In this framework, the *Plac1*-null transcriptome reflects disruption of regulatory nodes whose downstream effects evolve as the placenta matures and adapts to functional insufficiency.

Conservation of the Plac1 sequence provides mechanistic context for its role. Plac1 shares ~30% homology with the zona pellucida binding protein ZP3 [1,111], a member of a conserved family of extracellular matrix proteins that organize pericellular structures adjacent to the plasma membrane [111–113]. ZP3-like motifs are also present in multiple receptor-associated extracellular glycoproteins, including betaglycan and uromodulin [114]. Oocyte-enriched zona pellucida domain (ZPD) proteins (Oosp1–3) also share homology with Plac1 [115,116]. In Drosophila, ZPD proteins regulate epidermal cell shape and apical membrane–ECM interactions during embryogenesis [117], suggesting a conserved role in organizing membrane–cytoskeletal interfaces relevant to morphogenesis.

Specific Plac1-protein interactions further support a membrane-adjacent regulatory role. Plac1 interacts directly with desmoglein-2 (Dsg2), a desmosomal protein [118], implicating roles in cell–cell adhesion, polarity, migration, and invasion. Dsg2 can also localize to apical compartments in enterocytes [119], raising the possibility of non-canonical roles linking the actin-rich terminal web to cytoskeletal systems. This is notable given Plac1 localization near the apical syncytiotrophoblast brush border and the F-actin-rich terminal web [5]. In addition, Plac1 binds extracellular FGF7/FGFRIIIb to activate AKT signaling [120,121] and interacts with the proprotein convertase furin to influence invasion-related Notch/NICD/PTEN signaling [122]. When viewed together, these interactions converge on cytoskeletal regulation and Rho-linked signaling pathways, consistent with the dominant pathway-level features observed in our transcriptomic analyses.

Although these interactions were identified in cancer models, they remain informative for developmental physiology as well. Dsg2 is essential for cardiovascular development. Loss-of-function mutations are associated with cardiomyopathy and embryonic lethality in both humans and mice [123–125]. Notably, the *Plac1*-null transcriptome displayed enrichment for Dsg2-associated cardiomyopathy programs, i.e. dilated/arrhythmogenic cardiomyopathy, and both proteins are expressed in the developing myocardium. This convergence raises the possibility that Plac1 contributes to Dsg2-dependent cardiovascular function during critical developmental windows, a hypothesis supported by transcriptomic patterns but requiring future direct experimental validation.

This possibility is strengthened by the enrichment of sarcolemmal components among Plac1-regulated transcripts at E18.5 (GO Cellular Components; Supplemental Table S11) and a ~2.7-fold downregulation of Titin (*Ttn*) at E18.5 (Supplemental Table S6). Ttn spans half the length of the sarcomere from the Z-disk to the M-line, where it provides structural scaffolding, passive elasticity, and mechanosensory function [126]. Truncating *Ttn* variants are the most common genetic risk factor for peripartum cardiomyopathy and have been identified in women with preeclampsia [127,128], implicating sarcomeric vulnerability as a shared feature of hypertensive pregnancy-associated cardiac disease. Because Dsg2 insufficiency produces Z-disk defects and impaired sarcomeric force transmission, disruption of the Plac1–Dsg2 axis during cardiomyocyte maturation may compromise desmosome–sarcomere coupling, providing a plausible conceptual framework for cardiomyopathic features.

Consistent with this framework, and independent of transcriptomic inference, we unexpectedly observed concordant postnatal pathology in the last surviving Plac1 knockout male examined at 17 months of age. Although limited to a single animal and therefore not suitable for quantitative inference, this animal exhibited marked cardiomegaly with histologic features consistent with cardiomyopathic remodeling and pulmonary congestion secondary to cardiac dysfunction. (Supplementary Figure S9). While preliminary, this finding aligns with the observed placental enrichment of cardiovascular disease-associated pathways and known PLAC1–DSG2 interactions, and may reflect disruption of signaling pathways that are implicated in cardiovascular development, either through placental dysfunction or direct embryonic effects of Plac1 loss. Future studies examining larger cohorts and functional cardiac assessments will be required to define the reproducibility and developmental timing of this outcome.

The second mechanistic consideration involves Plac1 interactions with membrane-associated proteolytic systems, including furin. Furin is highly expressed in the syncytiotrophoblast, where it mediates proteolytic processing of substrates essential for placental differentiation and function, including the IGF1R and syncytins. Downregulation of the proprotein convertase Bace2 at E18.5 in Plac1-null placentas further suggests impaired capacity for regulated cleavage of membrane-associated substrates late in gestation (Supplemental Data, Table S6). Because furin participates in the maturation of multiple proteases within the secretory pathway, Plac1 loss may disrupt coordinated proteolytic processing through both transcriptional and post-translational mechanisms.

Whether analogous pathways operate in neural tissue, where Plac1 is expressed and protease-dependent processing of cell-surface proteins such as L1CAM is well established [129,130], remains a biologically plausible extension of these placental observations. L1CAM mutations are the most common cause of X-linked hydrocephalus, and Plac1 and L1cam reside in close genomic proximity. Notably, hydrocephalus associated with Plac1 mutant mice exhibits incomplete penetrance, analogous to L1cam-associated disease. The interaction of Plac1 with both furin and Dsg2 therefore places it at the intersection of membrane organization, cell–cell contact, and regulated proteolysis in the syncytiotrophoblast.

A key implication of these observations is that physiologically meaningful Plac1 interactions likely vary by cellular compartment, gestational timing, and trophoblast differentiation state. Consistent with this context-dependence, Plac1 immunostaining in human trophoblasts is diffuse in the first trimester and progressively localizes near the apical, microvillous membrane as gestation progresses [4,5]. Defining the determinants of Plac1 localization and interaction across developmental contexts remains an important area for future investigation.

The integration of manual gene-level curation with GO/KEGG/IPA analyses supports a coherent model of Plac1 function at the maternal–fetal interface. Plac1 loss likely impairs optimal establishment of the placental–maternal interface, predisposing it to ongoing nutrient and oxygen limitations and subsequent metabolic and oxidative stress. Disruption of plasma membrane signaling further compromises growth, remodeling, and immune tolerance. Because our transcriptomic window was restricted to E16.5–E18.5, events occurring earlier in placentation can only be inferred and will require targeted investigation.

Among IPA pathways, Rho GTPase signaling was the most consistently downregulated across both developmental ages, aligning with suppression of actin-based structures and membrane junctions. As a master regulator of cytoskeletal dynamics [131,132], Rho coordinates extracellular signal transduction governing proliferation, migration, polarity, vascularization, and immune tolerance. Disruption of Wnt, TGF-β, VEGF, HGF, IGF, SHH, BMP, and PDGF signaling, which all cross-talk with Rho-associated machinery [133–137], further underscores collapse of membrane-proximal signaling hubs. Dysregulated tetraspanins suggest perturbation of tetraspanin-enriched microdomains (TEMs), which organize integrins and growth-factor receptors essential for branching morphogenesis, angiogenesis, and ECM remodeling [138–142].

Plac1’s role extends beyond extraembryonic tissue [3]. Unsurprisingly, dysregulated pathways in the KO placenta were also enriched for modules relevant to fetal organ development where Plac1 expression has been demonstrated, including cardiovascular and neurodevelopmental programs, as well as kidney, lung, and musculoskeletal system. These observations align with epidemiological links between fetal growth restriction, prematurity, and developmental vulnerability [143,144], and are consistent with emerging concepts of placenta–brain and placenta–heart axes. Furthermore, metabolic alterations, including disrupted selenoamino acid metabolism and thyroid hormone handling, provide additional plausible mechanisms by which placental dysfunction may influence fetal neurodevelopment.

The relevance of the *Plac1-*null transcriptome to human pregnancy is further suggested by emergence of a PE-associated signaling signature beginning at E16.5 and strengthening over time. This timing approximates the human mid-to-late gestational window when clinical PE manifests. Additionally, dysregulated glycosylated fibronectin and reduced placental PLAC1 expression mirror findings in human PE and studies involving placental hypoxia [145–150]. Lastly, the *Plac1*-null transcriptome, like PE, is strongly associated with cardiovascular defects and orofacial clefts among other developmental defects [151], suggesting similar underlying mechanisms (See Table S24: IPA summary - “Diseases and Biological Processes”; downregulated genes). While unsupervised disease enrichment analyses identified significant vascular and endothelial diseases rather than PE, itself, this pattern suggests that the transcriptional changes reflect vascular and endothelial stress programs central to PE pathophysiology, without necessarily recapitulating the full disease-defining gene expression signature during this timeframe. Accordingly, the preeclampsia signal identified by IPA likely reflects convergence of cardiovascular and endothelial pathways associated with the disorder, rather than evidence of an established PE transcriptional state.

PE remains a leading cause of perinatal mortality and long-term morbidity [152–154], with approximately half of susceptibility attributable to genetic factors across maternal, paternal, and fetal contributions [155,156]. Future studies should include maternal phenotyping, earlier gestational windows, and cell-resolved approaches to define trajectories of PE-like progression. Inclusion of female heterozygous and knockout animals will be necessary to define sex-specific responses. Progressive lethality across generations [6], including among heterozygotes, suggests intergenerational or epigenetic contributions consistent with the DOHaD framework [157,158].

Finally, although this study focuses on the placenta, these findings parallel cancer biology. *PLAC1* is reactivated in multiple malignancies where it associates with invasive/EMT-like transitions [159,160], proliferative and angiogenic signaling [161,120,121], and immunosuppressive microenvironments that mirror maternal–fetal immune tolerance [162,163]. Combined with minimal PLAC1 expression in normal adult tissues, these parallels support a model in which PLAC1 participates in conserved developmental programs that can be co-opted during cancer-related disease progression [10,164,165].

## 4. Conclusions

Our findings position Plac1 as a critical upstream regulator of membrane-proximal signaling pathways required for placental structural integrity and homeostasis. Its absence disrupts cytoskeleton-linked regulatory networks, resulting in progressive placental stress and transcriptional changes consistent with placental insufficiency and preeclampsia-associated biology. Integration of gene-level expression with pathway-level analyses support a model in which Plac1 influences growth factor signaling, junctional organization, and vascular and immune adaptation through Rho GTPase–centered mechanisms. Together, these results identify Plac1 as a key determinant of placental function with implications for placenta-mediated developmental vulnerability. The functional role and mechanisms of action of Plac1 are likely context dependent, varying by cell type, tissue, and developmental stage. Delineating these mechanisms will require systematic investigation of Plac1 protein interactions, genetic variation, post-translational regulation, and downstream signaling networks.

## 5. Materials and Methods

### 5.1 Mutant mouse model

The entire *Plac1* open reading frame (aa 2–173) was deleted in murine ES cells (C57BL/6NTac) as part of the NIH Knockout Mouse Program (KOMP). Blastocysts were injected with the *Plac1*-null ES cells and chimera obtained. After germline transmission was achieved the mice were bred against a C57BL/6 background (Jackson Laboratories) as previously described [6]. For the studies described in this report, timed matings between hemizygous, *Plac1* mutant (knockouts) or wild type (WT) males and heterozygous *Plac1* females (Hets) were carried out. Pregnant females were sacrificed at E16.5 and E18.5 to obtain placental DNA in accordance with a protocol approved by the Institutional Animal Care and Use Committee (IACUC) of the University of South Florida-Morsani College of Medicine.

### 5.2 Genotyping and sex determination of mice

DNA was isolated from embryonic mouse tails using a DNeasy Blood and Tissue Kit (Qiagen). *Plac1* genotype was determined by PCR, using the primers:

5’-CCAATCATGTTCACCCACATTTCTAC-3 WT forward

5’-CCCTAAAAGAGCTATCATGGCATCT-3 Reverse

5’-GCAGCCTCTGTTCCACATACACTTCA-3 Neo universal forward

The cycling parameters were 94°C for 5 min followed by 10 cycles of 94°C for 15 s, 65°C for 30 s (decreased by 1°C at each repeat), and 72°C for 40 s; followed by 30 cycles of 94°C for 15 s, 55°C for 30 s, and 72°C for 40 s. PCR products were terminated with a final extension at 72°C for 5 min, then held at 4°C. A 1% agarose gel was used to visualize the generated wild type and mutant bands at 548 bp and 326 bp, respectively. Embryonic sex was determined by PCR using mouse SRY primers: 5’-TGGGACTGGTGACAATTGTC-3’ and 5’-GAGTACAGGTGTGCAGCTCT-3’ [12] to score for maleness. The cycling parameters were 95°C for 4.5 min followed by 33 cycles of 95°C for 35 s, 55°C for 1 min and 72°C for 1 min, and final extension 72°C for 5 min, then held at 4°C. A 1% agarose gel was used to visualize the generated SRY fragment at 402 bp.

### 5.3 Quantitative Real-time PCR

Placental RNA was extracted using the AllPrep DNA/RNA Mini Kit (Qiagen) as previously described [3,6]. Briefly, isolated placental RNA was used for quantitative real-time PCR. 2 ug of total RNA was used to synthesize the complementary cDNA with primer oligo dT and the SuperScript III First-Strand Synthesis SuperMix (Invitrogen) according to the manufacturer’s protocol. The RT-PCR reaction was performed using 2 uL cDNA with 1 uL 20X Taqman mouse, gene-specific mix (Applied Biosystems) targeting each of six selected genes (*Cer1 – Mm00515474_m1*; *Nppb* – Mm01255770_g1; *Elavl4* – Mm01263580_mH; *Zbtb4* – Mm07299415_g1; *Slca15* – Mm00558415_m1; Cnn1 – Mm00487032_m1), 10 uL TaqMan Universal PCR Master Mix (Applied Biosystems) and 7uL autoclaved RNAase free water for a total volume of 20 uL. The original assay catalog numbers are no longer retrievable from laboratory records and the assay IDs listed represent currently available equivalents confirmed to target the same transcripts with equivalent specificity. As an internal control, a second real-time PCR reaction was performed using 2 μl of a 1:10 diluted cDNA with 1 μl 20× Taqman 18S probe (Applied Biosystems, Assay ID Hs99999901_s1), 10 μl 2× Master Mix (Applied Biosystems), and 7 μl autoclaved RNAase free water for a total volume of 20 μl. Thermocycling conditions were as follows: 50°C, 2 min; 95°C, 10 min; and 40 cycles of 95°C for 15 s and 60°C for 1 min. RNA expression relative to 18s was calculated. A 7500 Real Time PCR System machine (Applied BioSystems) and 7500 System Software was used. Assays were performed in triplicate on duplicate WT and KO placentas. The original raw qRT-PCR data files were not available for re-analysis. Therefore, statistical testing could not be independently reproduced.

### 5.4 Microarray Analysis

Differential microarray analysis was carried out using the Agilent 4×44K mouse chip representing over 43,674 unique mouse transcripts as described by Carter, et al [166] and briefly summarized below.

#### 5.4.1 RNA Extraction, Target Labeling, Hybridization and Scanning

Total RNA extracted from the placentas of male WT and *Plac1*-null mice at E16.5 and E18.5 was extracted and purified using TriZol reagent (Invitrogen) per the manufacturer’s protocol. The quality and quantity of the preparations were assessed using an RNA 6000 Nano Lab-on-a-chip Kit with a 2100-Bioanalyzer system (Agilent Technologies).

Amplified cRNA labeled with Cyanine-3 CTP and Cyanine-5 CTP (Perkin-Elmer/NEN Life Sciences) was produced using a Fluorescent Linear Amplification Kit (Agilent Technologies) as specified by the manufacturer. The quality and size distribution of targets were determined by RNA 6000 Nano Lab-on-a-chip Assay (Agilent Technologies), and quantitated.

Fluorescent linear amplified cRNAs used in biological comparisons were then hybridized to Agilent 4×44K 60-mer oligo microarrays per the manufacturer’s instructions. Hybridized microarrays were washed according to the manufacturer’s protocol and scanned on an Agilent Technologies G2565AA Microarray Scanner System with SureScan technology.

#### 5.4.2 Data Processing and Statistical Analysis

Ratio data were extracted from scanned microarray images using Feature Extraction 5.1.1 software (Agilent Technologies). Dye-normalized, background-subtracted intensity and ratio data were exported to text and GEML-format files. Text output was originally processed using an application developed in-house (National Institute on Aging) to perform ANOVA analysis. Intensity values were filtered to remove values where probe error was greater than two times mean error and relative error was greater than 50%. Mean dye-swapped log(ratio) values were calculated, and mixed-model ANOVA was applied [166]. The potential for error variance was addressed by Bayesian adjustment to reduce false positives.

Subsequent to processing the original scan data in 2012 the processing software was published online as the interactive tool, ExAtlas [167] in 2015 and is continuously updated as gene annotations evolve. The original scan data were reprocessed (2026) in ExAtlas as described [167, 168]. A comprehensive all oligo matrix containing all probe features across all genotypes was extracted from ExAtlas. To improve annotation reliability and reduce probe redundancy, the all-oligo matrix was subjected to a two-step filtering process. Probes were filtered in two sequential steps to ensure retention of the highest quality and most reliable features. First, probes were filtered based on GenBank accession number priority. For any given gene symbol, probes associated with curated RefSeq mRNA accession numbers (NM_) were preferentially retained, and if they were present, all other accession types (XM_, AB_, AK_, etc.) were discarded. In cases where no NM_ accession was present, probes with predicted RefSeq accession numbers (XM_) were retained only if no other accession types were available, otherwise XM_ accessions were also discarded in favor of alternative accessions (e.g., AB_, AK_). Second, among the remaining probes, the single best-performing oligo per gene was selected based on the highest F-statistic value (lowest p-value) from a one-way ANOVA across genotypes, ensuring retention of the probe with the greatest discriminatory power. This two-step filtering process produced the best oligo matrix (Supplemental Table S1), which served as the basis for the statistical gene expression analysis Anova table (Supplemental Table S2) and all subsequent differential expression analyses between KO and WT at E16.5 and E18.5. The results were deposited in GEO (Accession - GSE308499). For datasets in which biological replication was limited, statistical significance was derived using the variance modeling framework implemented in ExAtlas, which estimates gene-level variance across the dataset rather than relying solely on within-group replication. Accordingly, results from these datasets are interpreted as exploratory and descriptive, and were used primarily to assess overall data quality, trends and concordance with replicate-supported datasets rather than to support independent statistical conclusions. Differentially expressed genes exhibiting at least a 1.5-fold change and an FDR < .05 were then subjected to KEGG, GO and Ingenuity Pathway Analysis (IPA) to sort them into physiologically relevant pathways and functions.

### 5.5 Transmission Electron Microscopy

To assess ultrastructural morphology, placental samples were fixed overnight in 2.5% glutaraldehyde in 0.1 M phosphate buffer (pH 7.4) at 4°C. Samples were washed in 0.1M Sodium Cacodylate buffer, pH 7.4. Post-fixation was performed in 1% osmium tetroxide in 0.1 M cacodylate buffer. After washing, the samples were then dehydrated in a series of graded ethanol concentration from 35% to 100%, followed by two washes in absolute acetone. The samples tissues were infiltrated and embedded in Embed812 resin. Ultrathin sections (80 nm) were mounted on copper grids and images captured by a Gatan Orius digital camera mounted on a JEOL 1400 electron microscope at the Microscopy and Cell Imaging Core at the University of South Florida.

## Supplementary Material

Supplement 1 Supplementary Tables
Processed Expression Data and Statistical Analyses
**Table S1.** Best-oligo-filtered gene expression matrix
**Table S2.** Gene expression ANOVA results
**Table S3.** Principal component analysis (PCA) output
**Table S4.** E16.5 downregulated genes
**Table S5.** E16.5 upregulated genes
**Table S6.** E18.5 downregulated genes
**Table S7.** E18.5 upregulated genes
Gene Ontology (GO) Enrichment Analyses
**Table S8.** GO analysis—E16.5 downregulated genes
**Table S9.** GO analysis—E16.5 upregulated genes
**Table S10.** GO analysis—E16.5 dysregulated genes
**Table S11.** GO analysis—E18.5 downregulated genes
**Table S12.** GO analysis—E18.5 upregulated genes
**Table S13.** GO analysis—E18.5 dysregulated genes
KEGG Pathway Enrichment Analyses
**Table S14.** KEGG analysis—E16.5 upregulated genes
**Table S15.** KEGG analysis—E16.5 dysregulated genes
**Table S16.** KEGG analysis—E18.5 downregulated genes
**Table S17.** KEGG analysis—E18.5 upregulated genes
**Table S18.** KEGG analysis—E18.5 dysregulated genes
Ingenuity Pathway Analysis (IPA)
**Table S19.** IPA canonical pathways—E16.5 downregulated genes
**Table S20.** IPA canonical pathways—E16.5 upregulated genes
**Table S21.** IPA canonical pathways—E18.5 downregulated genes
**Table S22.** IPA canonical pathways—E18.5 upregulated genes
**Table S23.** IPA comparison analysis of shared upstream regulators (E16.5 and E18.5; downregulated genes)
**Table S24.** IPA summary—E16.5 downregulated genes
**Table S25.** IPA summary—E18.5 downregulated genes
**Table S26.** IPA Upstream Regulators – E16.5 downregulated genes
**Table S27.** IPA Upstream Regulators – E16.5 upregulated genes
**Table S28.** IPA Upstream Regulators – E18.5 downregulated genes
**Table S29.** IPA Upstream Regulators – E18.5 upregulated genes
Supplementary Figures
**Figure S1.** Replicates of RT-qPCR gene assays with native scales
**Figure S2.** IPA comparison analysis of shared canonical pathways (E16.5 vs. E18.5; upregulated genes)
**Figures S3–S8.** Original electron micrographs corresponding to Figure 10A–F in the main manuscript
**Figure S9.** Gross and microscopic images depicting cardiomegaly in an adult KO male

## Figures and Tables

**Table 1.** Genes Exhibiting the Greatest Dysregulated at E16.5 and E18.5 (Positive and negative signage denote directionality; FDR < 0.05)

E16.5				E18.5			
Downregulated	Fold Change *(Linear)*	Upregulated	Fold Change *(Linear)*	Downregulated	Fold Change *(Linear)*	Upregulated	Fold Change *(Linear)*
***Plac1***	−73.063	*Igfbp1*	+15.174	***Plac1***	−63.915	*Clec2m*	+34.978
*Tff3*	−22.146	*Khdc1b*	+13.586	*Krt6a*	−45.446	*Cer1*	+30.584
*Gzmg*	−21.592	*Minar1*	+9.727	*Elavl4*	−23.774	*Lrfn2*	+27.511
*Angptl3*	−19.512	*Syt15*	+9.467	*Bhlhe22*	−21.984	*Spata21*	+23.768
*Gzmf*	−17.179	*Farp1*	+9.120	*Ntrk2*	−17.636	*1700027H10Rik*	+22.714
*Gzmc*	−16.634	*Ifi203*	+7.984	*Ceacam18*	−13.605	*Aldh1a3*	+21.817
*Vmn1r17*	−16.118	*Tcra*	+7.912	*Dio2*	−12.514	*Clec9a*	+21.632
*Ehd4*	−15.531	C730014E05Rik	+7.248	*Or51e2*	−11.479	*Pla2g4d*	+16.297
*Neurod4*	−14.458	*Gabrq*	+6.871	*Isx*	−11.061	*Ascl2*	+15.150
		9330162B11Rik	+6.634	*Gzmc*	−10.048	*Psmb9*	+13.993

**Table 2.** Genes Reciprocally Dysregulated at E16.5 and E18.5 (Positive and negative signage denote directionality; FDR < 0.05)

A. Dynamic Developmental Shift *(E16.5 Downregulated – to – E18.5 Upregulated)*			B. Dynamic Developmental Shift *(E16.5 Upregulated – to – E18.5 Downregulated)*		
Genes	E16.5 Fold Change *(Linear)*	E18.5 Fold Change *(Linear)*	Genes	E16.5 Fold Change *(Linear)*	E18.5 Fold Change *(Linear)*
*Asz1*	−2.412	+2.537	*Cnmd*	+2.715	−3.766
*Dnajb11*	−1.648	+1.682	*Crispld2*	+2.418	−2.093
*Dnajc3*	−1.834	+1.582	*Gpx3*	+2.748	−2.012
*Gm12618*	−1.892	+1.604	*Tfpi2*	+1.698	−1.550
*Gm773*	−3.337	+2.159	*Porcn*	+1.893	−2.205
*Noct*	−2.221	+1.749	*Gpnmb*	+1.584	−1.919
*Prl2c3*	−2.010	+1.549	*H6pd*	+2.003	−2.591
*Prl7a2*	−4.394	+1.938	*Siglecg*	+2.097	−2.017
*Prl8a8*	−1.710	+1.995	*Sphk1*	+2.080	−2.344
*Rab27a*	−1.797	+2.143	*Trnp1*	+1.667	−2.279
*Tmsb10*	−2.877	+1.703	*Tatdn2*	+2.430	−2.165
*Prom1*	−1.579	+1.558	*Spon1*	+2.820	−2.248
			*1700064E03Rik*	+3.458	−2.949

**Table 3.** Functional Classes of Downregulated Genes (non-inclusive)

**Brain development/function**	*Ntrk2, Elavl4, Gata4, Nog, Nefm, Negr1, Cnrip1, Syt12, Fzd6, Slc26a4. Slc6a15, Amigo2, Hes1*
**Solute transporters**	*Slc26a4, Slc10a6, Slc28a3, Slc2a10, Slc45a3, Slc1a6, Slc22a23, Slc22a4, Slc43a3, Slc1a5, Slc39a4, Slc44a3, Slc7a14, Slc7a6, Slc66a3, Slc27a3, Slc40a1, Slc26a2, Slc5a2, Slc6a14, Slc5a6, Slc8a1, Slc20a2, Slc19a2, Slc16a5, Slc7a10*
**Ion channels**	*Trpv6, P2rx4, P2rx1, Orai1, Kcnmb1, Kcnk5, Kcnd3, Trpc4, Ano1, Scnn1a, Cacna1g, Kcnmb2*
**GPCR signaling (receptors and mediators)**	*Adora1, F2rl1, Gcgr, Or51e2, Cysltr2, Tbxa2r, Gprc5a, Gprc5b, Gpr146, Mrgprf, Mrgprg, Rxfp1, Adgra2, Adgrl4, Lgr4*
**Plasma Membrane Signal Transduction**	*Wnt2, Tspan1, Tspan2, Tspan4, Tspan6, Tspan7, Tspan12, Pdgfb, Pdgfc, Smo*

**Table 4.** KEGG Analysis of Upregulated Genes

KEGG Term	E16.5 Fold Enrichment	E16.5 FDR	E18.5 Fold Enrichment	E18.5 FDR
Ribosome	6.36	4.16E-16	9.38	3.02E-45
Coronavirus Disease-COVID 19	4.87	3.26E-12	6.88	5.71E-36
Glycolysis/Gluconeoneogenesis	5.75	8.95E-04	---	---
Antigen processing/presentation	4.84	1.06E-03	---	---
Allograft rejection	5.55	1.60E-03	---	---
Graft versus host disease	5.55	1.60E-03	---	---
Type I diabetes mellitus	4.94	4.12E-03	---	---
Biosynthesis of amino acids	4.46	7.31E-03	---	---
Autoimmune thyroid disease	4.33	7.31E-03	---	---
Carbon metabolism	3.66	7.41E-03	---	---
Pentose phosphate pathway	6.51	7.41E-03	---	---
Viral myocarditis	3.82	1.42E-02	---	---
Amino and nucleotide sugar metabolism	5.67	1.76E-02	---	---
Phagosome	2.91	1.96E-02	---	---
Cell adhesion molecules	2.87	2.2E-02	---	---
HIF-1 signaling pathway	3.36	2.6E-02	---	---
VEGF signaling pathway	4.36	3.1E-02	---	---
Epstein-Barr virus infection	2.52	3.93E-02	---	---
Fructose and mannose metabolism	5.13	4.37E-02	---	---
Biosynthesis of nucleotide sugars	4.99	4.97E-02	4.63	4.98E-02
Protein Processing in ER	---	---	2.7	3.77E-02
Amoebiasis	---	---	3.09	4.98E-02
Cellular senescence	2.65	4.97E-02	---	---

**Table 5.** Canonical Pathways Associated with Cardiovascular and Brain Development

Cardiovascular Development	Pathway	E16.5		E18.5	
		p-value (−log)	Z-score	p-value (−log)	Z-score
	Rho GTPase Cycle	2.47	−5.099	5.81	−7.141
	Factors Promoting Cardiogenesis in Vertebrates	2.9	−2.887	2.31	−3.5
	Cardiac Hypertrophy Signaling (Enhanced)	1.38	−4.264	0.961	−5.112
	Myocardin Signaling Pathway	1.95	−2.333	4.78	−2.982
	Cardiomyocyte Differentiation via BMP Receptors	2.85	−2.236	3.3	−2.646
	Hey1 Signaling Pathway	1.13	-0.333	4.08	−2.4
	ABRA Signaling Pathway	2.71	−1.667	2.97	−1.941
	Nitric Oxide Signaling in the Cardiovascular System	0.489	−1.342	0.925	−2.333
Brain Development	Pathway	p-value (−log)	Z-score	p-value (−log)	Z-score
	Rho GTPase Cycle	2.47	−5.099	5.81	−7.141
	Myelination Signaling Pathway	1.21	−3.0	2.30	−3.286
	Synaptic Long-Term Depression	1.29	−1.897	0	−3.0
	L1CAM Interactions	1.9	−3.0	0.944	−3.162
	WNT/SHH Axonal Guidance Signaling Pathway	1.24	−2.333	2.31	−2.183
	Neurovascular Coupling Signaling Pathway	0.723	−1.667	1.1	−2.668

**Table 6.** Dysregulation of Fibronectin-associated Genes

Genes	E16.5		E18.5	
	Fold Change *(Linear)*	FDR	Fold Change *(Linear)*	FDR
*Fn1*	---	---	+1.949	.0007
*Galnt5*	+2.598	.0071	---	---
*Galnt6*	---	---	+2.582	.0033
*Galnt12*	+1.696	.0189	+2.644	0
*Galnt14*	+3.17	0	+3.214	0
*Galnt15*	+2.7	.0016	---	---
*B4galnt2*	---	---	+4.413	.0003
*St3gal3*	+2.1	.0315	+2.465	.0032
*St6gal1*	---	---	+1.561	.0293
*Mgat1*	+1.763	.0071	---	---

**Table 7.** Representative upstream regulators illustrating opposing predicted activation states of vascular and developmental signaling versus inflammatory/stress-associated pathways in *Plac1*-null placentas.

Downregulated Genes			Upregulated Genes		
Gene	E16.5 *(Z-score)*	E18.5 *(Z-score)*	Gene	E16.5 *(Z-score)*	E18.5 *(Z-score)*
*Vegf*	−5.761	−6.699	*Il1b*	+3.760	+5.334
*Hgf*	−3.427	−5.122	*Rela*	+2.433	+4.094
*Igf1*	−3.532	−3.854	*Hif1a*	+3.943	+4.557
*Tgfb1*	−2.356	−5.387	*Rictor*	−2.729	−6.328
*Fgf2*	−1.426	−3.529	*Larp1*	−5.475	−8.243
*Bmp4*	−3.904	−3.878	*Lats*	−2.121	−3.207
*Srf/Mrtfb*	−3.396/−3.514	−4.206/−5.25	*Stat3*	+1.672	+2.660
*Yap1*	−3.014	−4.353	*Map3k8*	+1.707	+2.652
*Tead2*	−2.236	−4.123	*Ikbke*	+2.626	+2.796
***Tnf***	−2.326	−4.324	***Tnf***	+5.264	+6.002
***Ifng***	−3.377	−4.755	***Ifng***	+4.457	+4.633

## Data Availability

The raw scan and processed data derived from the microarray studies was deposited to GEO and will be openly available *(*Accession – GSE308499*).* The curated microarray data summarized in this manuscript is included in the article and supplementary material. The original electron micrographs used in the manuscript are included in the supplementary material. All original contributions are contained within the article and supplementary material. Further inquiries can be directed to the corresponding author.
