# Transcriptomic Analysis of *Plac1* Ablation Reveals Broad Alterations in Signaling Pathways Essential for Prenatal Development and Overlap with a Preeclampsia-Associated Signature

**DOI:** 10.64898/2026.04.30.721637

**Published:** 2026-07-13

**Authors:** Suzanne Jackman, Xiaoyuan Kong, Yulan Piao, Alexei Sharov, Elin Lehrmann, Andrew Varshine, Ramaiah Nagaraja, David Schlessinger, Michael E. Fant

**Affiliations:** 1 Department of Pediatrics, University of South Florida, Morsani College of Medicine, Tampa, FL; 2 Laboratory of Genetics and Genomics, National Institute on Aging, National Institutes of Health, Baltimore, Maryland

**Keywords:** Plac1, placental development, Rho GTPase signaling, fetal growth restriction (FGR), birth defects, cardiovascular disease, preeclampsia, brain development, Developmental Origins of Health and Disease (DOHaD)

## Abstract

*Plac1* is an X-linked gene essential for placental and embryonic development. A knockout (KO) mouse model was used to define placental gene expression changes associated with Plac1 loss at E16.5 and E18.5 using gene expression microarray. Genes exhibiting at least a 1.5-fold change and FDR < 0.05 were considered significant. At E16.5, 717 genes were downregulated and 796 upregulated in KO placentas relative to wild type (WT), whereas at E18.5, 1121 genes were downregulated and 1151 upregulated. Subsets of highly and uniquely dysregulated genes were examined by gene-level curation, alongside systems-level analyses (GO, KEGG, and IPA) applied to the full DEG datasets. Downregulated genes were enriched for Rho GTPase-mediated and actin cytoskeleton-based processes, as well as membrane-associated signaling pathways with established roles in placental and embryonic development, vascular function, and branching morphogenesis. Overlap with pathways and molecular features associated with preeclampsia was also observed. In contrast, upregulated genes reflected, in part, immune activation and oxidative stress responses. These findings represent an important exploratory, hypothesis-generating analysis and provide a biologically coherent framework for understanding how Plac1 loss may be associated with placental dysfunction and pregnancy-related disease.

## 1. Introduction

*Plac1* is an X-linked gene that maps to a chromosomal locus previously shown to be important for placental and embryonic development [1]. Its expression is particularly high in the placenta, hence its name **Plac**enta-specific **1**, as the placenta was initially thought to be the principal site of its expression [1,2]. Subsequent studies, however, demonstrated that Plac1 is also expressed throughout the developing embryo, albeit at substantially lower levels than in the placenta, suggesting a spatially broader role during prenatal development. In adult animals, expression diminishes to essentially undetectable levels [3]. The human *PLAC1* gene encodes a putative protein of 212 amino acids, whereas the mouse ortholog encodes a 173-amino-acid protein with substantial sequence conservation [1]. The protein is predicted to exist as a membrane-associated or extracellular peptide, with a predicted transmembrane domain near the N-terminus. Within the placenta, PLAC1 expression is restricted primarily to trophoblast lineages and is closely linked to differentiation of the syncytiotrophoblast [1,2,4]. In humans, immunohistochemical studies have localized PLAC1 near the apical cytosolic region of the differentiated syncytiotrophoblast in proximity to the maternal-facing microvillous membrane surface [5], consistent with a role at membrane-associated interfaces important for trophoblast function.

An essential role for Plac1 in placental and embryonic development has been demonstrated in a mutant mouse model [6]. Placentas in *Plac1*-null mice exhibit placentomegaly with an enlarged and disorganized junctional zone (JZ), characterized by spongiotrophoblast (SpT) hyperplasia and associated with mild fetal growth restriction. Consistent with preferential paternal X chromosome inactivation in murine extraembryonic tissue [7,8], genetic analyses revealed that placentas derived from maternal (X^m-^X) heterozygotes (Hets) were phenotypically similar (but not identical) to knockout (KO) placentas whereas paternal (XX^p-^) Hets were phenotypically indistinguishable from wild type (WT). Further analysis suggested that the paternal *Plac1* allele is not completely inactivated, with approximately 10–15% residual activity remaining, providing some degree of functional activity in the X^m-^X Het not observed in the KO. The changes in the placental phenotype were later supported by Muto, et al [9], using a mutant mouse model derived using a different targeting vector and bred against a different mouse strain. By employing Lentivirus-mediated *Plac1* expression, they were also able to show that Plac1 rescue failed to reverse the overgrowth of the SpT layer but did ameliorate changes in the labyrinth, pointing to temporal aspect(s) of Plac1 function. These observations provide compelling evidence that indicate the loss of Plac1 produces a robust developmental phenotype involving the architecture and functional organization of the placenta.

A role for Plac1 beyond the placenta has also been suggested. Surviving *Plac1*-null males and maternal heterozygous females exhibit increased susceptibility to postnatal hydrocephalus, supporting the possibility that Plac1 contributes to developmental processes in the embryo proper as well as in extraembryonic tissues [3]. Together, these studies support the view that Plac1 participates in shared biological processes required for normal placental development, fetal growth, and pregnancy maintenance. However, the molecular pathways associated with the placental consequences of Plac1 loss remain incompletely defined.

PLAC1 has also attracted interest outside of developmental biology due to its reactivation in multiple human cancers, where it has been associated with processes such as proliferation, migration, invasion, and immune modulation [10]. These cancer-related observations do not establish equivalence between trophoblast biology and malignancy, but they do suggest that PLAC1 participates in cellular programs involving membrane-associated signaling, tissue remodeling, and regulated cell–cell interactions. In the placenta, such processes are central to trophoblast differentiation, maternal–fetal interface organization, vascular adaptation, and pregnancy maintenance. Thus, defining transcriptional changes associated with Plac1 loss in the placenta may provide insight into developmental pathways in which Plac1 participates and may also help clarify why PLAC1-linked biology is relevant across developmental and disease contexts.

In the present study, we re-examined placental transcriptomic data generated from *Plac1*-null and wild-type mouse placentas at E16.5 and E18.5, a late-gestational interval during which the mutant placental phenotype is well established. The objective was not to define a complete mechanistic cascade downstream of Plac1, but rather to identify transcriptional patterns and signaling pathways associated with Plac1 loss in the placenta. To this end, we combined manual curation of highly and uniquely dysregulated genes with GO, KEGG, and IPA pathway analyses to capture both gene-level and systems-level alterations. This integrated strategy was designed to preserve biological context while identifying broader pathway-level themes.

Given the limited biological replication available from this legacy *Plac1* mutant colony, the findings are interpreted as exploratory and hypothesis-generating. Nonetheless, they support a model in which *Plac1* ablation is associated with broad alterations in signaling pathways essential for prenatal development, including those overlapping with molecular features associated with preeclampsia, and provide a biologically coherent framework for investigating how Plac1 loss may contribute to placental dysfunction and pregnancy-related disease within the Developmental Origins of Health and Disease framework.

## 2. Results

### 2.1 Developmental dynamics of placental growth in Plac1 mutants

**Figure 1.**
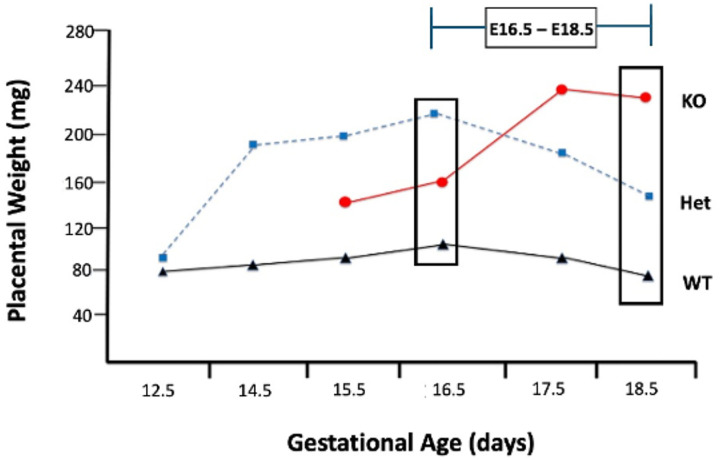
Growth trajectories of placentas from *Plac1* mutants compared to WT placentas. Placental weights were determined throughout gestation representing WT, X^m-^X, and KO mice and presented in graphical form summarizing previously published data [6]. Each point represents the mean of age-specific samples for each genotype (n = 1–10 per data point). WT and KO data include both male and female placentas.

This analysis was focused on the period of pregnancy when the growth trajectory of *Plac1*-null placentas exhibit maximal divergence from WT placentas. Placentas associated with *Plac1*-null embryos and maternal Hets (X^m-^X) exhibit placentomegaly and a disorganized junctional zone (JZ) [6]. Placental weights of X^m-^X Hets diverge from KO littermates between E16.5 and E18.5, likely because the paternal allele escapes complete inactivation. As we previously reported [6] and summarize in Figure 1, X^m-^X Het placental weight peaks at E16.5 and decreases slightly thereafter. By contrast, the weight of *Plac1*-null placentas continues to accelerate until E17.5 and then plateaus. These observations informed our decision to examine the developmental span bordered by E16.5 and E18.5.

### 2.2 Gene Expression Microarray Analysis

The Agilent 4×44k gene chip was used to identify *Plac1*-dependent gene expression at E16.5 and E18.5. KO male placentas were compared to WT male placentas in duplicate samples. At E16.5, 717 known or putative genes were downregulated and 796 genes were upregulated at least 1.5-fold (FDR < 0.05) in KO placentas compared to WT. Similarly, at E18.5, 1121 genes were downregulated and 1151 genes were upregulated. (See supplemental materials, Tables S1–S7, for processed data and complete gene lists).

**Figure 2.**
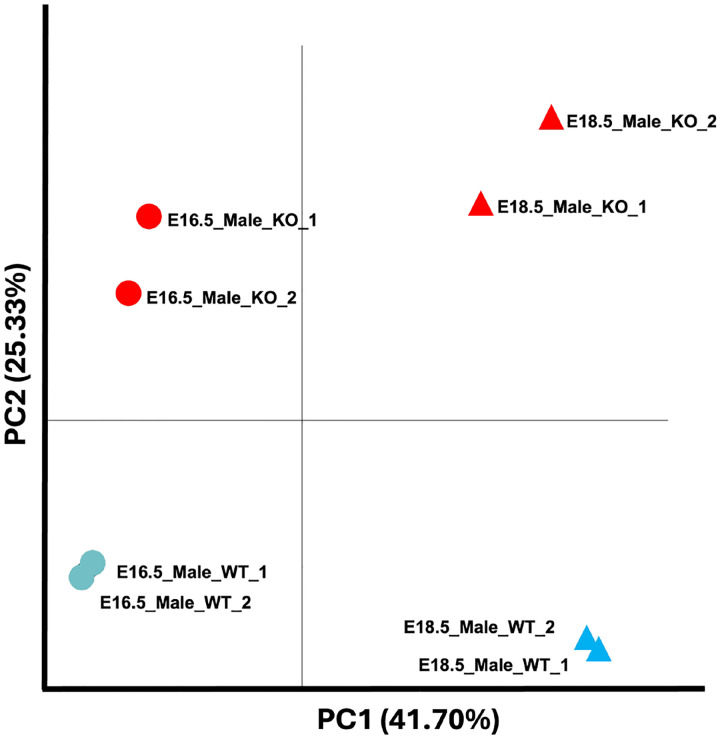
PCA of normalized expression data from E16.5 and E18.5 male placentas (WT and KO). PC1, and PC2 explain [41.70%] and [25.33%] of the variance, respectively. PC1 separates E16.5 vs E18.5, PC2 separates WT vs KO. Points are plotted by age, genotype, and replicate. Color = genotype (WT = blue, KO = red); Shape = age (E16.5 = circles, E18.5 = triangles)

Principal Component Analysis (PCA) (Supplemental Table S3) revealed that the first two components explained 67.03% of the variance (PC1: 41.7%, PC2: 25.33%). Visual inspection of PC1 and PC2 (Figure 2) indicates that PC1 primarily separated samples by gestational age (E16.5 versus E18.5, whereas PC2 distinguished genotypes (WT from KO).

**Figure 3.**
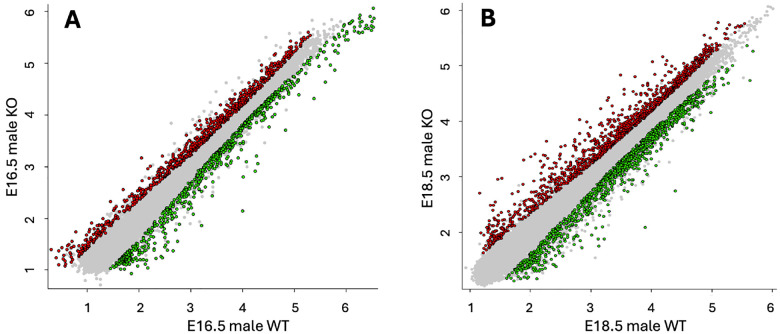
KO vs WT scatter plots by developmental stage. Pairwise scatter plots compare KO (y-axis) versus WT (x-axis) log10 expression within **(A)** E16.5 males (replicate means) and **(B)** E18.5 males (replicate means). Each point represents a gene/probe; the diagonal line indicates y = x (equal expression in KO and WT). Red points represent upregulated genes in the KO; green points represent downregulated genes in the KO; grey points represent no significant dysregulation, based on ≥1.5-fold change and FDR < 0.05.

KO versus WT scatter plots are shown for each age group (Figure 3). Most genes lie near the diagonal reference line, indicating broadly similar expression between KO and WT. Colored points denote differentially expressed genes (DEGs) meeting our threshold (≥1.5-fold change, FDR < 0.05). Red indicates upregulated genes in the *Plac1* KO and green indicates downregulated KO genes. Grey points indicate genes not significantly dysregulated. Axes show log_10_ expression values.

**Figure 4.**
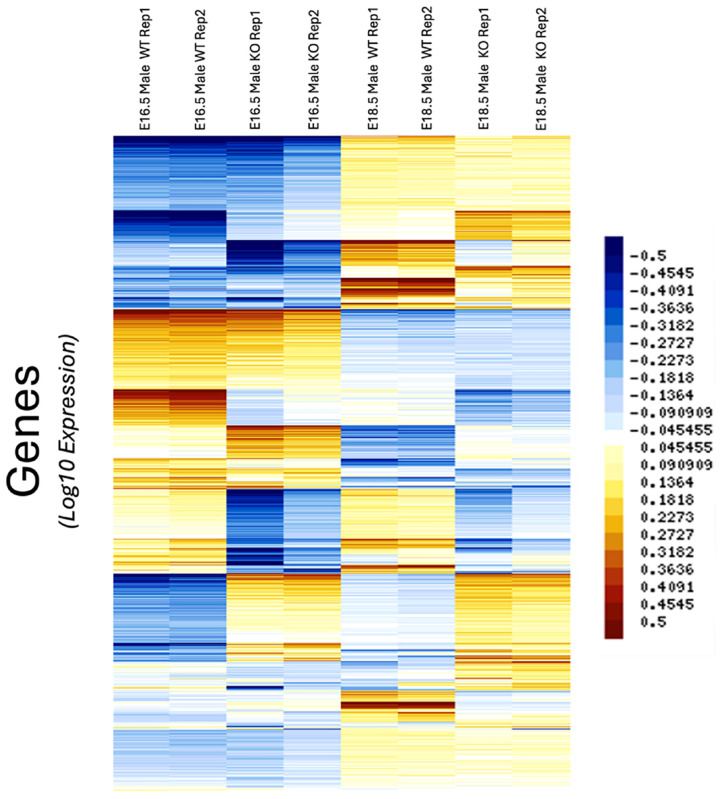
Heatmap of differentially expressed genes across samples. Heatmap displays relative gene expression patterns for differentially expressed genes identified at each developmental stage. For visualization, log_10_-transformed expression values were row-centered such that each gene’s mean expression across all samples equals zero. Color indicates expression relative to that gene’s mean level (blue, lower than average; yellow/brown, higher than average). Values were not variance-scaled. Genes (rows) are ordered by hierarchical clustering.

The heatmap of differentially expressed genes is shown in Figure 4. Expression values are visualized, with genes (rows) ordered by hierarchical clustering. qRT-PCR assays were performed at the time of the original microarray analysis for a limited panel of six genes at E18.5, with the number of assays constrained by colony attrition. These data are provided in Supplementary Figure S1 as qualitative directional observations only and not as independent validation of the microarray results.

### 2.3 Gene-level Curation of Highly and Reciprocally Dysregulated Genes

We initially applied a targeted manual curation strategy to select subsets of differentially expressed genes (DEGs) to determine whether these transcripts could provide physiological context for the transcriptomic changes observed in the *Plac1*-null placenta. This approach was intended to be illustrative and hypothesis-generating rather than exhaustive, and to identify biologically coherent patterns that might otherwise be obscured when transcripts are considered only at the level of pathway enrichment.

Two complementary criteria were used for this purpose. First, we examined DEGs exhibiting the greatest magnitude of fold-change at each developmental stage (E16.5 and E18.5), irrespective of whether they were upregulated or downregulated (Table 1). This subset was considered because large-amplitude expression changes may reflect prominent biological responses within the *Plac1*-null placental environment. Second, we identified an additional subset of genes exhibiting reciprocal dysregulation across developmental stage, that is, genes downregulated at E16.5 and upregulated at E18.5 or, conversely, upregulated at E16.5 and downregulated at E18.5 (Table 2). These reciprocally regulated genes were considered because they may reflect evolving adaptive, compensatory, or decompensatory responses as placental dysfunction progresses during late gestation. (See Supplemental Tables S4–S7 for complete gene lists).

Considered collectively, the highest-magnitude DEGs at each time point (Table 1) clustered within several broad functional categories. These included genes associated with trophoblast structural integrity, membrane-associated signaling, cytoskeletal regulation, vascular development and endothelial interaction, immune modulation, and metabolic or stress-responsive processes. Top-ranked downregulated genes included *Plac1* itself and genes associated with immune/trophoblast-interface signaling (*Gzmg, Gzmf, Gzmc*) [11–13], epithelial/cytoskeletal or adhesion-related programs (*Krt6a, Tff3, Ceacam18*) [14–16], membrane trafficking and receptor-mediated communication (*Ehd4, Ntrk2, Or51e2*) [17–21] vascular/endocrine-metabolic support (*Angptl3, Dio2, Isx*) [22–26], and developmental or neurodevelopmental annotations (*Elavl4*) [27]. Although *Neurod4*, *Vmn1r17*, and *Bhlhe22* were among the top-ranked downregulated transcripts, they lack convincing evidence for placental expression or function and were therefore excluded from consideration.

In contrast, upregulated genes were more often associated with immune/inflammatory signaling (*Tcrg-C, Ighm, Clec2m, Clec9a*) [28–30], metabolic or lipid-remodeling adaptation (*Aldh1a3, Pla2g4d*) [31, 32], and altered differentiation-, signaling-, or cellular remodeling-associated programs (*Minar1, Farp1, Ascl2, Cer1, Robo1, Syt15, Lrfn2*) [33–41]. Poorly characterized RIKEN or predicted transcripts were noted but not considered further. Interpreted at the level of functional clusters rather than as isolated gene-level effects, these patterns are broadly consistent with disruption of processes required for placental organization and maintenance and are generally concordant with the observed phenotype of *Plac1* mutant placentas.

The subset of reciprocally dysregulated genes (Table 2) provided an additional temporal perspective on the *Plac1*-null transcriptome. Genes shifting from reduced expression at E16.5 to increased expression at E18.5 included factors associated with ER/protein homeostasis (*Dnajb11*, *Dnajc3*) [42], vesicular trafficking or membrane-associated regulation (*Rab27a*, *Prom1*) [43, 44] and endocrine/placental lactogen-related support (*Prl2c4*, *Prl7a2*, *Prl8a8*) [45–47]. Conversely, genes elevated at E16.5 but reduced by E18.5 included factors associated with extracellular matrix or anti-angiogenic regulation (*Cnmd*, *Spon1*) [48–50], immune modulation (*Crispld2* [51], *Siglecg*) [52,53], oxidative/ER-redox stress handling (*Gpx3*, *H6pd*) [54–56], and lipid/signaling or developmental remodeling pathways (*Sphk1*, *Porcn*) [57–60].

These observations are consistent with altered temporal coordination of placental support programs in the absence of Plac1 and identify physiologically relevant transcriptional themes whose collective functional annotations align with the known *Plac1*-null placental phenotype. Importantly, this analysis does not assign causality or direct regulatory hierarchy to individual genes or pathways, nor does it establish mechanism. However, manual curation of these uniquely expressed gene subsets provides a complementary layer of analysis that preserves gene-level biological context when integrated with the pathway enrichment analyses.

#### 2.3.1 Functional Classes of DEGs

Examination of the DEGs also revealed a striking enrichment for membrane-associated receptors, solute transporters, and ion channels suggesting broad disruption of membrane-linked signaling and transport functions in the *Plac1*-null placenta. (Table 3). Notably, many of these genes are also annotated to brain development, consistent with shared placental and neurodevelopmental regulatory programs and with the CNS pathology observed in *Plac1* knockout mice [3].

#### 2.3.2 Systems-level and Pathway Analyses: GO (Gene Ontology), KEGG (Kyoto Encyclopedia of Genes and Genomes), Ingenuity Pathway Analysis (IPA)

We next performed systems-level analyses to understand how Plac1 loss converges on broader biological processes. To this end, we used GO, KEGG, and IPA tools to identify shared functional themes and regulatory networks disrupted in the *Plac1*-null placenta.

##### GO Analysis

**Figure 5.**
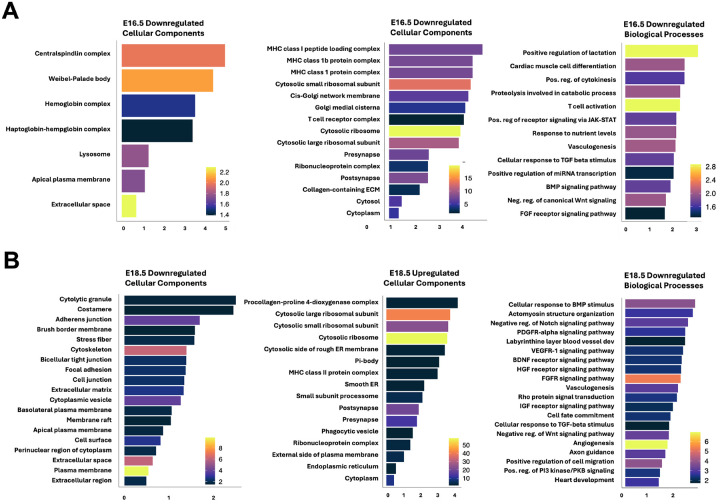
Developmental Stage-Specific Dysregulation of Cellular Components and Biological Processes in KO Placentas. Bars represent GO terms significantly enriched among the dysregulated genes. The X-axis shows log_2_ (Enrichment), indicating fold enrichment relative to WT controls. The Y-axis lists individual GO terms. Bar colors correspond to −log_10_(FDR), with warmer colors (yellow/orange) indicating higher statistical significance and cooler colors (blue/purple/black) indicating lower significance. **Panel A:** E16.5, showing downregulated (left) and upregulated (middle) Cellular Components, and downregulated Biological Processes (right). **Panel B:** E18.5, showing downregulated (left) and upregulated (middle) Cellular Components, and downregulated Biological Processes (right). * Note – For visualization purposes, the number of BPs and CCs were selectively limited to a maximum of 20 by reducing implied functional redundancy.

GO enrichment demonstrated predominant downregulation of cellular components (CC) associated with the membrane–cytoskeletal interface, including the apical, brush border plasma membrane, lysosomes, extracellular matrix, adherens and tight junctions, and actin-based structures at both E16.5 and E18.5 (Figure 5 A, B; see Supplemental Tables S8–S11 for complete GO term lists). These findings are consistent with the reported localization of PLAC1 to membranous compartments, particularly near the apical trophoblast surface. Additionally, enrichment of spindle-associated structures suggested possible effects on cytokinesis-related programs, while involvement of Weibel–Palade body-associated terms at E16.5 suggested altered endothelial-associated signaling. Given the role of Weibel–Palade bodies in the regulated release of von Willebrand factor, angiopoietin 2, and endothelin 1, genes associated with this compartment are particularly relevant to hemostasis, vascular tone, and angiogenesis, [61–63].

In contrast, upregulated CC terms reflected stress-adaptive and compensatory responses, most notably enrichment of translational machinery, ribonucleoprotein complexes, and ER/Golgi-associated compartments, particularly at E18.5. These changes are consistent with increased translational load, secretory activity, and proteostasis demand under nutrient and oxidative stress. Immune-related components also showed significant enrichment, represented by the MHC class I complex at E16.5 followed by MHC class II complex and phagocytic vesicles at E18.5. Additional enrichment of extracellular matrix and synapse-annotated compartments, likely reflecting vesicle trafficking and cytoskeletal remodeling, further supported broad alteration of membrane dynamics and cell–cell communication.

Downregulated GO biological process (BP) analysis revealed enrichment of growth factor and developmental signaling pathways, including BMP, FGF, Wnt, TGFβ, and Jak–STAT, all of which have established roles in placental development [64–66]. In addition, dysregulated genes in the placental dataset were enriched for GO annotations related to embryonic organogenesis, particularly cardiovascular pathways. These annotations likely reflect shared developmental signaling programs used across embryonic tissues, including placenta, rather than direct evidence of organspecific developmental pathology within placental tissue. GO BP enrichment for upregulated genes at both E16.5 and E18.5 was represented by generalized immune, stress, and metabolic response terms that overlapped extensively with KEGG and IPA pathway annotations described below and is presented in full in the Supplementary Data (Tables S9 and S11).

##### KEGG Analysis

**Figure 6.**
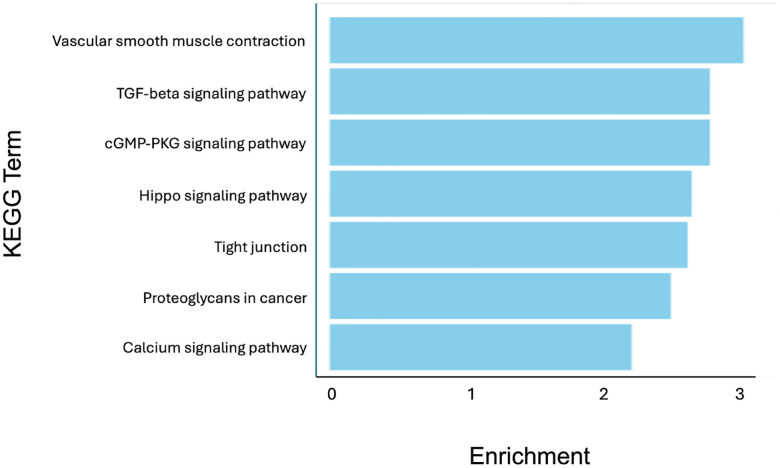
Fold enrichment of KEGG terms represented by downregulated genes at E18.5. Bars represent KEGG pathways significantly enriched among downregulated genes at E18.5. The X-axis shows fold enrichment, and the Y-axis lists individual KEGG terms (FDR < 0.05).

KEGG analysis at E18.5 reinforced these findings, identifying enrichment among downregulated genes for pathways related to vascular smooth muscle contraction, calcium and cGMP–PKG signaling, tight junction integrity, TGFβ signaling, and Hippo pathway components (Figure 6; see Supplementary Tables S12–S15 for complete KEGG term lists). These pathways are broadly associated with vascular regulation, cytoskeletal organization, trophoblast proliferation, and structural stability, and are therefore consistent with altered placental remodeling and maternal–fetal interface function. No significant downregulated KEGG terms were identified at E16.5.

Conversely, KEGG terms enriched among upregulated genes were dominated by cellular stress, metabolic adaptation, and immune activation, including Ribosome, Protein Processing in the Endoplasmic Reticulum, Glycolysis/Gluconeogenesis, Phagosome, and Antigen Processing and Presentation (Table 4; Supplementary Tables S13 and S15). The viral pathway annotation, *Coronavirus Disease – COVID 19*, likely reflects heightened innate and adaptive immune signaling rather than pathogen-specific responses.

##### Ingenuity Pathway Analysis (IPA)

Broadening our systems-level interpretation of the *Plac1*-null transcriptome, we next applied IPA analysis. Unlike gene-level fold-change comparisons, IPA integrates both the directionality of gene expression changes and curated regulatory relationships among pathway components to predict pathway activity states. Canonical pathways were therefore evaluated using the IPA activation Z-score, which estimates whether the collective expression pattern of pathway-associated genes is more consistent with activation or inhibition relative to random expectation. This approach enables functional inference at the pathway level and complements the enrichment-based GO and KEGG analyses described above.

**Figure 7.**
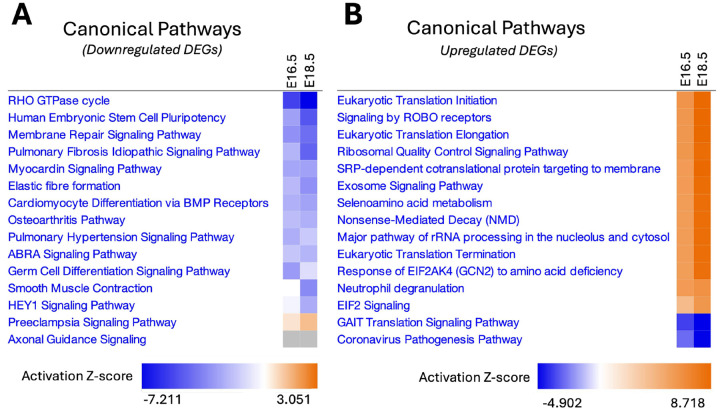
Heatmaps Depicting Developmental Stage-Specific Dysregulation of IPA Canonical Pathways in KO Placentas **Panel A.** Canonical Pathways associated with Downregulated genes at E16.5 and E18.5. **Panel B.** Canonical Pathways associated with Upregulated genes at E16.5 and E 18.5. Color intensity reflects activation Z-scores: blue indicates negative Z-scores (predicted inhibition), and orange indicates positive Z-scores (predicted activation). Pathways filtered for absolute Z-scores > 2 and Benjamini–Hochberg adjusted p-values < 0.05 at one or more developmental stages and ranked by absolute Z-score.

IPA “Comparison Analysis” identified Rho GTPase signaling as the most significantly downregulated pathway across both developmental stages (Figure 7A; see Supplementary Tables S16–S19 for complete pathway lists), along with myocardin signaling, elastic fiber formation, membrane repair, cardiomyocyte differentiation via BMP receptors, and embryonic stem cell pluripotency pathways [67–69]. Upregulated IPA pathways were highlighted by predicted activation of translational control, ribosomal quality control, nonsense-mediated decay, EIF2/GCN2-mediated integrated stress response and antioxidant metabolism, consistent with chronic nutrient and/or oxidative stress [70–72] (Figure 7B; see Supplemental Table S20 and Figure S2). Suppression of GAIT and coronavirus pathogenesis pathway annotations may reflect altered inflammatory and antiviral-response regulatory programs, including impaired translational restraint of cytokine-associated signaling [73,74]. Collectively, GO, KEGG, and IPA analyses converge on a model in which loss of *Plac1* disrupts membrane-proximal, actin-cytoskeletal, and vascular regulatory networks, accompanied by progressive placental stress, immune dysregulation, and functional decline.

#### 2.3.3 Downregulation of Developmental Signaling Annotated to Brain and Cardiovascular Systems

Several canonical pathways associated with downregulated DEGs in the *Plac1*-null placenta were annotated to developmental processes involving embryonic organ systems, including cardiovascular and neural development. (See Supplemental Tables for downregulated IPA summaries: “Diseases and Biological Functions”, S21 and S22). These annotations likely reflect conserved developmental signaling pathways that are reused across multiple biological contexts, including placental development, rather than evidence of organ-specific developmental programs within placental tissue. Accordingly, these pathway enrichments are interpreted as identifying shared developmental signaling networks that may be relevant to the broader *Plac1*-null phenotype.

Pathways relevant to the structural and functional development of the cardiovascular system were disproportionately represented and strengthened over the course of gestation, as indicated by their activation Z-scores and B-H-adjusted p-values (Table 5). We interpret these enrichments as reflecting altered shared regulatory signaling networks rather than direct evidence of cardiovascular development within placental tissue. Although specific cardiovascular disorders were not an identified feature of our model, embryos and surviving mice were not systematically examined for defects that may have been present but otherwise not apparent. Notably, mice carrying the *Plac1*-null and X^m-^X genotypes exhibited increased fetal or perinatal lethality that ultimately approached complete lethality after multiple generations.

Pathways annotated to brain development were also enriched, consistent with shared developmental signaling programs and with the increased risk of hydrocephalus observed in adult Plac1 KO and maternal heterozygous mice. Rho GTPase signaling pathways play major roles in CNS development and function. Additionally, enrichment of axonal guidance pathways may be relevant in this context because these pathways contribute to branching morphogenesis [75–77], a fundamental process in placental development as well as in other embryonic tissues, including lung, kidney, and brain.

#### 2.3.4 Transcriptomic Overlap with Preeclampsia-Related Pathways and Molecular Features

**Figure 8.**
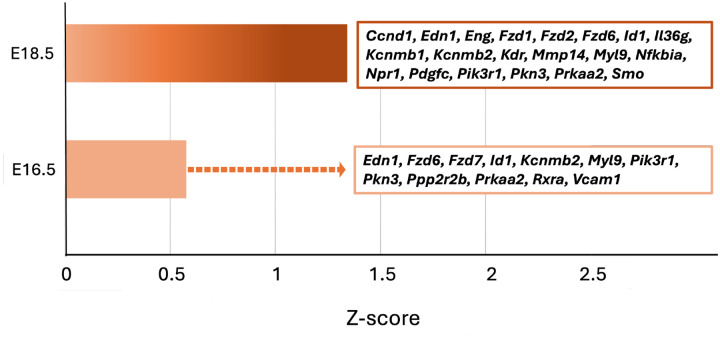
Transcriptomic Overlap with Preeclampsia-Related Pathway Features in *Plac1*-null Placentas PE-associated pathways at each developmental age were identified using downregulated DEGs exhibiting at least a 1.5-fold decrease in expression compared to WT placentas. **E16.5** - Solid light orange color. (Z-score = 0.58, FDR = 0.18) **E18.5** - Dynamic orange fill depicting strengthening activation and significance level over time. (Z-score = 1.34, FDR = 0.02) **Text boxes:** DEGs populating the canonical PE pathway at each developmental age (Supplemental Tables S21 and S22). The vertical axis denotes gestational age. The horizontal axis denotes positive Z-scores. The dashed line (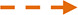) represents the dynamic shift toward activation from E16.5 to E18.5

A notable feature of the *Plac1*-null transcriptome was its overlap with pathways and molecular features reported for preeclampsia (PE) and related placental stress states. DEGs contributing to the PE-associated canonical pathway were identified at E16.5 and strengthened over time (Figure 8). At E18.5, this pathway reached statistical significance (FDR = 0.02) and a positive dynamic shift in the activation Z-score (0.58 to 1.34), suggesting increased concordance between the *Plac1*-null expression profile and curated PE-associated molecular features. However, because the Z-score did not reach the conventional threshold for confident pathway activation (Z-score = 2.0), these findings are best interpreted as transcriptomic overlap with PE-related biology rather than evidence that Plac1 loss produces a PE-like disease state.

Unsupervised disease–gene enrichment analyses of the E18.5 downregulated DEGs provided additional context for this pathway-level observation. DisGeNET analysis identified vascular diseases, hypertensive diseases, and vascular inflammation among enriched disease classes (q = 0.01466 – 0.04304), as well as thrombosis (q = 0.04180). By contrast, preeclampsia itself was identified only in the expanded OMIM analysis and did not reach significance after multiple-testing correction (p = 0.043, q = 0.1618; OR = 2.05). Taken together, these results are consistent with convergence between the *Plac1*-null transcriptomic profile and vascular, endothelial, inflammatory, and placental stress pathways relevant to PE, rather than defining a categorical PE transcriptional state.

A separate group of upregulated DEGs was also associated with fibronectin processing and glycosylation, molecular features relevant to PE biomarker biology. Elevated maternal serum glycosylated fibronectin (GlyFn) has been reported as a predictive marker for PE [78, 79]. In the present dataset, placental *Fn1* expression was increased together with several genes involved in post-translational glycosylation, including members of the *Galnt* family, *B4galnt2*, *St3gal3*, *St6gal1*, and *Mgat4a* (Table 6). The presence of *St6gal1* is notable in this context because GlyFn assays rely on recognition of α2,6-sialylated epitopes. These observations suggest that the *Plac1*-null placenta exhibits transcriptional changes involving fibronectin-associated expression and glycosylation pathways. However, altered GlyFn production, secretion or maternal serum biomarker levels were not assessed in this study.

Importantly, maternal blood pressure, proteinuria, circulating biomarkers, and other clinical features of PE were not assessed in the pregnant females from which these placentas were collected. Therefore, the present findings should not be interpreted as demonstrating PE in the *Plac1*-null model. Rather, they indicate that Plac1 loss is associated with transcriptional changes that overlap with molecular pathways implicated in PE and placental dysfunction. Future studies incorporating maternal phenotyping, expanded placental sampling, and direct measurement of PE-related biomarkers will be necessary to determine whether the transcriptomic overlap observed here corresponds to functional or clinical manifestations of PE-related placental disease.

#### 2.3.5 Predicted Upstream Regulator Patterns Are Concordant with Developmental and Stress-Associated DEG Signatures

To extend the pathway-level analyses, IPA “upstream regulator” (UR) analysis was used to identify predicted regulatory patterns associated with the downregulated and upregulated DEG datasets at E16.5 and E18.5. Because IPA upstream regulator analysis infers potential regulatory activity from the expression patterns of curated downstream target genes, these results should be viewed as computational predictions rather than direct evidence of regulator activation or inhibition.

Among downregulated DEGs, the filtered UR results (Z-score ≥ 2; FDR < 0.05) included multiple regulators associated with vascular development, growth-factor signaling, cytoskeletal organization, and developmental programs, including Vegf/Vegfa, Hgf, Igf1, Tgfb1, Fgf2, Bmp4, Srf/Mrtfb, Yap1, and Tead2 (Supplemental Tables S23, S25). These predicted patterns were broadly concordant with the pathway-level enrichment of membrane-associated signaling, Rho GTPase/cytoskeletal regulation, vascular adaptation, and developmental signaling pathways described above. Thus, the upstream regulator results provide an additional computational summary consistent with altered expression of gene networks linked to placental growth, structural organization, and maternal–fetal interface function.

In contrast, the upregulated DEG datasets included predicted upstream regulators associated with inflammatory, cytokine, hypoxia-associated, and stress-responsive gene-expression patterns, including Il1b, Rela, Hif1a, Il6, Map3k8, Ikbke, Tnf, and Ifng. These results were consistent with the enrichment of stress- and immune-associated pathways among upregulated genes. Importantly, several URs, including Tnf and Ifng, appeared in both downregulated and upregulated DEG analyses with different predicted activation states. This pattern reflects differences in the downstream target-gene subsets represented within each DEG group rather than uniform activation or inhibition of the regulator itself.

Together, these predicted upstream regulator patterns were consistent with the broader transcriptomic findings. Downregulated DEGs were associated with developmental, vascular, growth-factor, and cytoskeletal regulatory signatures, whereas upregulated DEGs were associated with cytokine-, hypoxia-, and stress-associated signatures. These findings complement the GO, KEGG, and IPA canonical pathway analyses and are best viewed as hypothesis-generating computational summaries of the DEG patterns. Complete upstream regulator results for upregulated and downregulated gene sets are provided in Supplementary Tables S23–S26.

### 2.4 Electron Microscopy (EM) of Plac1-null Placentas

**Figure 9.**
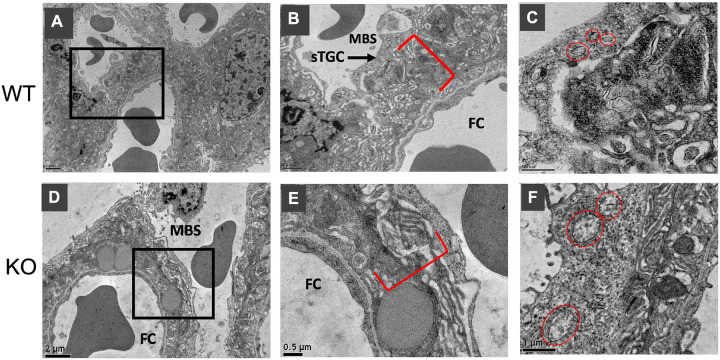
Transmission electron micrographs of the trilaminar interhemal region of E18.5 WT and KO placentas. **WT Panels:** The inset in panel **9A** shown at higher magnification in panel **9B**, depicts the trilaminar interhemal barrier separating the fetal capillaries (FC) from the maternal blood space (MBS). Sinusoidal trophoblast giant cells (sTGC; black arrow) are in direct contact with the MBS. A compact, well-defined region containing the syncytiotrophoblast (SynT) layers is indicated by the red brackets. Panel **9C** shows numerous small, well-defined mitochondria within an sTGC (red circles). **KO Panels:** Panels **9D** and **9E** show a similar interhemal region in the KO placenta. The SynT region appears more disordered and less compact. Panel **9F** depicts several enlarged mitochondria with poorly defined cristae (red circles). Scale bars = 0.5–2.0 μm.

TEM examination of placentas provided visual context for the molecular changes identified in the transcriptomic analyses. In the fields examined, structural differences were observed that were consistent with altered membrane organization, cytoskeletal regulation, and stress-response pathways. In an E18.5 WT placenta (Figure 9A–C), the labyrinth interhemal region displayed a well-organized trilaminar architecture. Sinusoidal trophoblast giant cells (sTGCs) lined the maternal blood space (MBS), and the syncytiotrophoblast (SynT) region formed an ordered layer overlying the basement membrane, followed by endothelial cells lining the fetal capillaries (FC). At higher magnification, mitochondria appeared small and relatively uniform, with discernible cristae. By contrast, in the *Plac1*-null placenta (Figure 9D–F), the corresponding interhemal region appeared less compact and less regularly organized in the field examined. The SynT region appeared less ordered, and enlarged mitochondria with less clearly defined cristae were observed in KO sTGCs.

These features are consistent with the broader transcriptomic pattern of altered membrane-associated signaling, cytoskeletal organization, and stress-response pathways in the *Plac1*-null placenta. However, because this analysis was limited to one placenta per genotype, these observations are presented as illustrative. Additional samples, systematic regional sampling, and quantitative ultrastructural analysis will be required to determine whether these features are reproducible and statistically associated with Plac1 loss. Source EM images are provided in Supplementary Figures S3–S8.

## 3. Discussion

The present study provides an exploratory transcriptomic analysis of the *Plac1*-null placenta during late gestation and identifies coordinated changes in gene expression patterns associated with multiple biological processes important for placental organization and function. By integrating targeted gene-level curation of highly and reciprocally dysregulated genes with GO, KEGG, and IPA analyses, we identified recurrent functional themes involving membrane-associated signaling, cytoskeletal organization, vascular and maternal–fetal interface regulation, immune modulation, and stress-responsive pathways. These transcriptional alterations occur in the context of the established *Plac1*-null placental phenotype and involve systems known to be important for normal placental development and pregnancy maintenance. Importantly, they provide a biologically coherent framework for understanding how the absence of Plac1 may be associated with disruption of coordinated regulatory programs relevant to placental dysfunction that evolve as the placenta matures and adapts. Despite the limited biological replication, the internal consistency of the dataset, reflected in clear separation by genotype and gestational age and convergence across independent pathway analyses, is further supported by its concordance with the established *Plac1*-null placental phenotype and with ultrastructural observations providing illustrative context. Because our transcriptomic window was restricted to E16.5–E18.5, alterations occurring earlier in placentation will require targeted investigation of those timeframes.

Among the pathway-level analyses, membrane-associated signaling, actin cytoskeletal regulation, and vascular-related processes were prominent features of the *Plac1*-null transcriptome. These systems are closely interconnected in the placenta, where trophoblast organization, maternal–fetal interface integrity, and labyrinthine vascular adaptation depend on coordinated signaling across membrane-proximal and cytoskeletal networks. The observed transcriptional changes in these functional categories are therefore consistent with disturbance of biological systems that are known to contribute to placental architecture and exchange function. However, because the present study is based on transcriptomic associations rather than direct functional testing, these findings do not demonstrate that Plac1 directly regulates any individual pathway or signaling node. Rather, they suggest that loss of Plac1 occurs in association with broad alteration of regulatory programs that converge on processes essential for late gestational placental maintenance.

Conservation of the Plac1 sequence provides suggestive mechanistic context for its role. Plac1 shares ~30% homology with the zona pellucida binding protein ZP3 [1,80], a member of a conserved family of extracellular matrix proteins that organize pericellular structures adjacent to the plasma membrane [80–82]. ZP3-like motifs are also present in multiple receptor-associated extracellular glycoproteins, including betaglycan and uromodulin [83]. Oocyte-enriched zona pellucida domain (ZPD) proteins (Oosp1–3) also share homology with Plac1 [84, 85]. In Drosophila, ZPD proteins regulate epidermal cell shape and apical membrane–ECM interactions during embryogenesis [86], suggesting a conserved role in organizing membrane–cytoskeletal interfaces relevant to morphogenesis.

Specific Plac1-protein interactions further support a membrane-adjacent regulatory role. Plac1 interacts directly with desmoglein-2 (Dsg2), a desmosomal protein [87], implicating roles in cell–cell adhesion, polarity, migration, and invasion. Dsg2 can also localize to apical compartments in enterocytes [88], raising the possibility of non-canonical roles linking the actin-rich terminal web to cytoskeletal systems. This is notable, given Plac1 localization near the apical syncytiotrophoblast brush border and the F-actin-rich terminal web [5]. In addition, Plac1 binds extracellular FGF7/FGFRIIIb to activate AKT signaling [89, 90] and interacts with the proprotein convertase furin to influence invasion-related Notch/NICD/PTEN signaling [91]. When viewed together, these interactions converge on cytoskeletal regulation and Rho-linked signaling pathways, consistent with the dominant pathway-level features observed in our transcriptomic analyses.

Although these interactions were identified in cancer models, they remain informative for developmental physiology as well. Dsg2 is essential for cardiovascular development. Loss-of-function mutations are associated with cardiomyopathy and embryonic lethality in both humans and mice [92–94]. Notably, the *Plac1*-null transcriptome displayed enrichment for Dsg2-associated cardiomyopathy programs, i.e. dilated/arrhythmogenic cardiomyopathy, and both proteins are expressed in the developing myocardium. This convergence raises the possibility that Plac1 contributes to Dsg2-dependent cardiovascular function during critical developmental windows, a hypothesis supported by transcriptomic patterns but requiring future direct experimental validation.

This possibility is strengthened by a 2.7-fold downregulation of Titin (*Ttn*) at E18.5 (Supplemental Table S6). Ttn spans half the length of the sarcomere from the Z-disk to the M-line, where it provides structural scaffolding, passive elasticity, and mechanosensory function [95]. Truncating *Ttn* variants are the most common genetic risk factor for peripartum cardiomyopathy and have been identified in women with preeclampsia [96, 97], implicating sarcomeric vulnerability as a shared feature of hypertensive pregnancy-associated cardiac disease. Because Dsg2 insufficiency produces Z-disk defects and impaired sarcomeric force transmission, disruption of the Plac1–Dsg2 axis during cardiomyocyte maturation may compromise desmosome–sarcomere coupling, providing a plausible conceptual framework for cardiomyopathic features.

As a separate anecdotal observation, we unexpectedly observed concordant postnatal pathology in the last surviving *Plac1* knockout male examined at 17 months of age. Although limited to a single animal and therefore not suitable for quantitative inference, this animal exhibited marked cardiomegaly with histologic features consistent with cardiomyopathic remodeling and pulmonary congestion indicative of secondary cardiac dysfunction. (Supplementary Figure S9). While preliminary, this finding aligns with the observed placental enrichment of cardiovascular diseaseassociated pathways and known PLAC1–DSG2 interactions, and may be consistent with altered signaling pathways implicated in cardiovascular development, either through placental dysfunction or direct embryonic effects of Plac1 loss. Future studies examining larger cohorts and functional cardiac assessments will be required to define the reproducibility and developmental timing of this outcome.

The second mechanistic consideration involves Plac1 interactions with membrane-associated proteolytic systems, including furin. Furin is highly expressed in the syncytiotrophoblast, where it mediates proteolytic processing of substrates essential for placental differentiation and function [98, 99]. Downregulation of the proprotein convertase Bace2 at E18.5 in *Plac1*-null placentas further suggests a reduced capacity for regulated cleavage of membrane-associated substrates late in gestation (Supplemental Table S6). Because furin contributes to the maturation of multiple proteases within the secretory pathway, loss of Plac1 may be associated with broader alterations in proteolytic processing through both transcriptional and post-translational mechanisms.

A notable feature of the *Plac1*-null phenotype is the increased risk of postnatal hydrocephalus. Mutations in the *L1CAM* gene represent the most common cause of X-linked hydrocephalus and its chromosomal locus lies in close proximity to the *PLAC1* locus. [100]. In addition, hydrocephalus in *Plac1* mutant mice exhibits incomplete penetrance, paralleling L1cam-associated disease [101]. Whether furin-dependent pathways operate in neural tissues, where Plac1 is also expressed and protease-mediated processing of cell surface proteins such as L1CAM is well established, remains a plausible extension of these findings. The interaction of Plac1 with both furin and Dsg2 therefore places it at the intersection of membrane organization, cell–cell interactions, and regulated proteolysis in the syncytiotrophoblast.

A key implication of these observations is that physiologically meaningful Plac1 interactions likely vary by cellular compartment, gestational timing, and trophoblast differentiation state. Consistent with this context-dependence, Plac1 immunostaining in human trophoblasts is diffuse in the first trimester and progressively localizes near the apical, microvillous membrane as gestation progresses [4,5]. Defining the determinants of Plac1 localization and interactions across developmental contexts remains an important area for future investigation.

Among IPA pathways, Rho GTPase signaling was among the most consistently downregulated canonical pathways and provides a plausible convergence point linking membrane-associated perturbations to cytoskeletal and structural placental programs. Rho-family GTPases coordinate extracellular signal transduction with actin organization, polarity, adhesion, migration, and vascular remodeling [102, 103]. Signaling pathways that interact with Rho-associated machinery, including Wnt, TGFβ, VEGF, HGF, IGF, SHH, BMP, and PDGF, have established roles in placental and developmental biology [104 – 108]. Dysregulated tetraspanins (Tspans) in our dataset may further indicate altered tetraspanin-enriched microdomains, which organize integrins and growth factor receptors relevant to branching morphogenesis, angiogenesis, and ECM remodeling [109–112].

Dysregulated pathways in the KO placenta were also enriched for annotations relevant to fetal organ development where Plac1 expression has been demonstrated, including cardiovascular and neurodevelopmental programs, as well as kidney, lung, and musculoskeletal system. These observations align with epidemiological links between fetal growth restriction, prematurity, and developmental vulnerability [113,114], and are consistent with emerging concepts of placenta–brain and placenta–heart axes. Furthermore, metabolic alterations, including disrupted selenoamino acid metabolism and thyroid hormone handling, provide additional plausible mechanisms by which placental dysfunction may influence fetal neurodevelopment.

A further interpretive feature of the *Plac1*-null transcriptome was the emergence of transcriptional changes overlapping with pathways and gene signatures that have been associated with preeclampsia. These associations were most evident at E18.5, where pathway analyses identified increased representation of vascular, endothelial, inflammatory, and stress-responsive programs that are also implicated in placental dysfunction associated with preeclampsia. In addition, dysregulation of genes related to fibronectin processing and glycosylation is notable given prior reports linking glycosylated fibronectin to preeclampsia risk. Additionally, IPA disease-annotation analyses (Supplemental Tables S21, S22) identified cardiovascular and developmental categories that overlap with features reported in association with preeclampsia-related pregnancy complications, particularly cardiovascular defects [115,116]. These findings indicate that the transcriptomic consequences of Plac1 loss include patterns that overlap with molecular features reported in preeclampsia and related placental stress states. Viewed collectively, these preeclampsia-related findings are best interpreted as supportive of broader convergence between the *Plac1*-null phenotype and pathways associated with placental dysfunction, while remaining hypothesis-generating and requiring further validation in appropriately powered experimental or clinical studies.

PE remains a leading cause of perinatal mortality and long-term morbidity [117–119], with approximately half of susceptibility attributable to genetic factors across maternal, paternal, and fetal contributions [120, 121]. Future studies should include maternal phenotyping, earlier gestational windows, and cell-resolved approaches to define PE-related molecular trajectories. Inclusion of female heterozygous and knockout animals will also be necessary to define sex-specific responses. Furthermore, the progressive lethality observed across generations [6], including among heterozygotes, suggests intergenerational or epigenetic contributions consistent with the DOHaD framework [122, 123].

Taken together, these findings support the view that the *Plac1*-null placenta exhibits a transcriptional profile consistent with broad disturbance of coordinated placental support systems rather than isolated alteration of individual genes or pathways. These processes are tightly integrated during late gestation and are essential for maintaining placental structure and maternal–fetal interface function. In this context, the *Plac1*-null transcriptome is most appropriately interpreted as defining a framework of molecular perturbations associated with an established placental phenotype, rather than as demonstrating direct mechanistic control by Plac1 over each affected pathway.

A central limitation of the present study is the limited biological replication inherent to this legacy dataset. The final DEG and pathway analyses were based on two biological replicates per genotype at each developmental stage. While the variance-modeling framework implemented in ExAtlas provides a structured approach for estimating gene-level variance across the dataset, it does not replace additional independent biological observations. Accordingly, the present findings should be interpreted as an exploratory, hypothesis-generating transcriptomic framework that identifies coordinated expression patterns and pathway-level associations accompanying Plac1 loss, rather than as definitive evidence of direct mechanistic regulation.

In this context, during preparation of this manuscript, Moreno-Irusta et al. [124] reported a contemporaneous, independent mechanistic analysis of PLAC1 function in rat and human trophoblast systems, including its interaction with furin and roles in trophoblast differentiation. These findings provide independent convergence for several principal features identified in the present study, particularly the involvement of PLAC1 in membrane-associated signaling and trophoblast functional regulation. Conversely, the present study extends those observations by identifying systems-level transcriptional alterations across the intact placenta, placing PLAC1-associated biology within the broader context of maternal-fetal interface function and pregnancy-related disease pathways. Together, these complementary levels of biological organization provide a more integrated and coherent framework for understanding PLAC1 function across cellular, tissue, and developmental scales.

Finally, although this study focuses on the placenta, these findings parallel observations in cancer biology. *PLAC1* is reactivated in multiple malignancies where it associates with invasive/EMT-like transitions [125, 126], proliferative and angiogenic signaling [127, 89, 90], and immunosuppressive micro-environments that mirror maternal–fetal immune tolerance [128, 129]. Combined with minimal PLAC1 expression in normal adult tissues, these parallels support a model in which PLAC1 participates in conserved developmental programs that can be co-opted during cancer-related disease progression [10,130,131].

## 4. Conclusions

In summary, Plac1 loss is associated with coordinated transcriptomic changes in the lategestation placenta, highlighted by altered placental developmental programs, reduced membrane-associated/Rho GTPase and actin-cytoskeletal signaling, and activation of immune, metabolic, and stress-response pathways. Together, gene-level curation and GO, KEGG, and IPA analyses are consistent with a model of developmental disequilibrium in the *Plac1*-null placenta, rather than disruption of a single linear pathway. These findings provide an exploratory, hypothesis-generating framework for future studies defining how Plac1 contributes to placental function, fetal developmental vulnerability, and pregnancy-related disease biology.

## 5. Materials and Methods

### 5.1 Mutant mouse model

The entire *Plac1* open reading frame (aa 2–173) was deleted in murine ES cells (C57BL/6NTac) as part of the NIH Knockout Mouse Program (KOMP). Blastocysts were injected with the *Plac1*-null ES cells and chimera obtained. After germline transmission was achieved the mice were bred against a C57BL/6 background (Jackson Laboratories) as previously described [6]. For the studies described in this report, timed matings between hemizygous, *Plac1* mutant (knockouts) or wild type (WT) males and heterozygous *Plac1* females (Hets) were carried out. Pregnant females were sacrificed at E16.5 and E18.5 to obtain placental RNA in accordance with a protocol approved by the Institutional Animal Care and Use Committee (IACUC) of the University of South Florida-Morsani College of Medicine.

### 5.2 Genotyping and sex determination of mice

DNA was isolated from embryonic mouse tails using a DNeasy Blood and Tissue Kit (Qiagen). *Plac1* genotype was determined by PCR, using the primers:

**Table:** 

5’-CCAATCATGTTCACCCACATTTCTAC-3	WT forward
5’-CCCTAAAAGAGCTATCATGGCATCT-3	Reverse
5’-GCAGCCTCTGTTCCACATACACTTCA-3	Neo universal forward

The cycling parameters were 94°C for 5 min followed by 10 cycles of 94°C for 15 s, 65°C for 30 s (decreased by 1°C at each repeat), and 72°C for 40 s; followed by 30 cycles of 94°C for 15 s, 55°C for 30 s, and 72°C for 40 s. PCR products were terminated with a final extension at 72°C for 5 min, then held at 4°C. A 1% agarose gel was used to visualize the generated wild type and mutant bands at 548 bp and 326 bp, respectively. Embryonic sex was determined by PCR using mouse SRY primers: 5’-TGGGACTGGTGACAATTGTC-3’ and 5’-GAGTACAGGTGTGCAGCTCT-3’ [12] to score for maleness. The cycling parameters were 95°C for 4.5 min followed by 33 cycles of 95°C for 35 s, 55°C for 1 min and 72°C for 1 min, and final extension 72°C for 5 min, then held at 4°C. A 1% agarose gel was used to visualize the generated SRY fragment at 402 bp.

### 5.3 Microarray Analysis

Differential microarray analysis was carried out using the Agilent 4×44K mouse chip representing over 43,674 unique mouse transcripts as described by Carter, et al [132] and briefly summarized below.

#### 5.3.1 RNA Extraction, Target Labeling, Hybridization and Scanning

Total RNA extracted from the placentas of male WT and *Plac1*-null mice at E16.5 and E18.5 was extracted and purified using TriZol reagent (Invitrogen) per the manufacturer’s protocol. The quality and quantity of the preparations were assessed using an RNA 6000 Nano Lab-on-a-chip Kit with a 2100-Bioanalyzer system (Agilent Technologies).

Amplified cRNA labeled with Cyanine-3 CTP and Cyanine-5 CTP (Perkin-Elmer/NEN Life Sciences) was produced using a Fluorescent Linear Amplification Kit (Agilent Technologies) as specified by the manufacturer. The quality and size distribution of targets were determined by RNA 6000 Nano Lab-on-a-chip Assay (Agilent Technologies), and quantitated.

Fluorescent linear amplified cRNAs used in biological comparisons were then hybridized to Agilent 4×44K 60-mer oligo microarrays per the manufacturer’s instructions. Hybridized microarrays were washed according to the manufacturer’s protocol and scanned on an Agilent Technologies G2565AA Microarray Scanner System with SureScan technology.

#### 5.3.2 Data Processing and Statistical Analysis

Ratio data were extracted from scanned microarray images using Feature Extraction 5.1.1 software (Agilent Technologies). Dye-normalized, background-subtracted intensity and ratio data were exported to text and GEML-format files. Text output was originally processed using an application developed in-house (National Institute on Aging) to perform ANOVA analysis. Intensity values were filtered to remove values where probe error was greater than two times mean error and relative error was greater than 50%. Mean dye-swapped log(ratio) values were calculated, and mixed-model ANOVA was applied [132]. The potential for error variance was addressed by Bayesian adjustment to reduce false positives.

Subsequent to initial processing of the original scan data in 2012, the analytical platform was published online as the interactive tool ExAtlas [133] in 2015 and has been continuously updated as gene annotations evolve. The original scan data were reprocessed in 2026 using ExAtlas as previously described [133,134]. A comprehensive all-oligo matrix containing all probe features across genotypes was extracted from ExAtlas. To improve annotation reliability and reduce probe redundancy, the matrix was subjected to a two-step filtering process designed to retain the highest-quality and most reliable features. First, probes were filtered based on GenBank accession priority. For any given gene symbol, probes associated with curated RefSeq mRNA accession numbers (NM_) were preferentially retained, and all other accession types (XM_, AB_, AK_, etc.) were discarded if NM_ accessions were present. In cases where no NM_ accession was available, probes with predicted RefSeq accession numbers (XM_) were retained only if no alternative accession types were present; otherwise XM_ probes were discarded in favor of other accessions (e.g., AB_, AK_). Second, among the remaining probes, a single best-performing oligo per gene was selected based on the highest F-statistic value (lowest p-value) from a one-way ANOVA across genotypes, ensuring retention of the probe with the greatest discriminatory power. This filtering process generated the best-oligo matrix (Supplemental Table S1), which served as the basis for construction of the ANOVA table (Supplemental Table S2) and all subsequent differential expression analyses between KO and WT placentas at E16.5 and E18.5. Female samples were excluded from the ANOVA due to lack of replication, and all subsequent analyses were restricted to male placental samples.

Statistical significance was derived using the variance-modeling framework implemented in ExAtlas, which estimates gene-level variance across the dataset rather than relying solely on gene-specific within-group replication. Because the final DEG analysis was based on two biological replicates per genotype at each developmental stage, the resulting gene lists are interpreted as exploratory and hypothesis-generating. Differentially expressed genes exhibiting at least a 1.5-fold change and an FDR < 0.05 were subjected to KEGG, GO, and Ingenuity Pathway Analysis (IPA) to identify functionally relevant pathways. All data have been deposited in GEO (accession: GSE308499).

### 5.4 Transmission Electron Microscopy

To assess ultrastructural morphology, placental samples were fixed overnight in 2.5% glutaraldehyde in 0.1 M phosphate buffer (pH 7.4) at 4°C. Samples were washed in 0.1M Sodium Cacodylate buffer, pH 7.4. Post-fixation was performed in 1% osmium tetroxide in 0.1 M cacodylate buffer. After washing, the samples were then dehydrated in a series of graded ethanol concentration from 35% to 100%, followed by two washes in absolute acetone. The tissues were infiltrated and embedded in Embed812 resin. Ultrathin sections (80 nm) were mounted on copper grids and images captured by a Gatan Orius digital camera mounted on a JEOL 1400 electron microscope at the Microscopy and Cell Imaging Core at the University of South Florida.

## Supplementary Material

Supplement 1

The following supplementary materials are available online for transparency and secondary analyses.

Supplementary Tables

Processed Expression Data and Statistical Analyses

- **Table S1.** Best-oligo-filtered gene expression matrix
- **Table S2.** Gene expression ANOVA results
- **Table S3.** Principal component analysis (PCA) output
- **Table S4.** E16.5 downregulated genes
- **Table S5.** E16.5 upregulated genes
- **Table S6.** E18.5 downregulated genes
- **Table S7.** E18.5 upregulated genes

Gene Ontology (GO) Enrichment Analyses

- **Table S8.** GO analysis—E16.5 downregulated genes
- **Table S9.** GO analysis—E16.5 upregulated genes
- **Table S10.** GO analysis—E18.5 downregulated genes
- **Table S11.** GO analysis—E18.5 upregulated genes

KEGG Pathway Enrichment Analyses

- **Table S12.** KEGG analysis—E16.5 downregulated genes (no significant Terms identified).
- **Table S13.** KEGG analysis—E16.5 upregulated genes
- **Table S14.** KEGG analysis—E18.5 downregulated genes
- **Table S15.** KEGG analysis—E18.5 upregulated genes

Ingenuity Pathway Analysis (IPA)

- **Table S16.** IPA canonical pathways—E16.5 downregulated genes
- **Table S17.** IPA canonical pathways—E16.5 upregulated genes
- **Table S18.** IPA canonical pathways—E18.5 downregulated genes
- **Table S19.** IPA canonical pathways—E18.5 upregulated genes
- **Table S20.** IPA comparison analysis of shared canonical pathways (E16.5 and E18.5; Upregulated genes)
- **Table S21.** IPA summary—E16.5 downregulated genes
- **Table S22.** IPA summary—E18.5 downregulated genes
- **Table S23.** IPA Upstream Regulators – E16.5 downregulated genes
- **Table S24.** IPA Upstream Regulators – E16.5 upregulated genes
- **Table S25.** IPA Upstream Regulators – E18.5 downregulated genes
- **Table S26.** IPA Upstream Regulators – E18.5 upregulated genes

Supplementary Figures

- **Figure S1.** Historical qRT-PCR plots for selected genes from the original microarray analysis
- **Figure S2.** IPA comparison analysis of shared canonical pathways (E16.5 vs. E18.5; upregulated genes)
- **Figures S3–S8.** Original electron micrographs corresponding to Figure 9A–F in the main manuscript
- **Figure S9.** Gross and microscopic images depicting cardiomegaly in an adult KO male

## Figures and Tables

**Table 1.** Genes Exhibiting the Greatest Dysregulation at E16.5 and E18.5

E16.5				E18.5			
Downregulated	Fold Change	Upregulated	Fold Change	Downregulated	Fold Change	Upregulated	Fold Change
***Plac1***	73.063	*Khdc1b*	13.586	***Plac1***	63.915	*Clec2m*	34.978
*Tff3*	22.146	*Tcrg-C*	12.397	*Krt6a*	45.446	*Cer1*	30.584
*Gzmg*	21.592	*Minar1*	8.980	*Elavl4*	23.774	*Lrfn2*	27.504
*Angptl3*	19.512	*Ighm*	8.527	*Bhlhe22*	21.984	*D430019H16Rik*	25.562
*Gzmf*	17.179	*9330162B11Rik*	8.406	*Ntrk2*	17.636	*Spata21*	23.768
*Gzmc*	16.634	*Gm4793*	7.556	*Ceacam18*	13.605	*1700027H10Rik*	22.714
*Vmn1r17*	16.118	*Robo1*	7.256	*Dio2*	12.514	*Aldh1a3*	21.817
*Ehd4*	15.531	C730014E05Rik	7.249	*Or51e2*	11.482	*Clec9a*	21.632
*Neurod4*	14.461	*Syt15*	7.186	*Isx*	11.061	*Pla2g4d*	16.297
*Isx*	14.385	Farp1	6.976	*Gzmc*	10.048	*Ascl2*	15.150

(Fold-Change = Linear Scale; FDR < 0.05)

**Table 2.** Genes Reciprocally Dysregulated at E16.5 and E18.5

A. Dynamic Developmental Shift *(E16.5 Downregulated – to – E18.5 Upregulated)*			B. Dynamic Developmental Shift *(E16.5 Upregulated – to – E18.5 Downregulated)*		
Genes	Fold Change E16.5 *(Downregulated)*	Fold Change E18.5 *(Upregulated)*	Genes	Fold Change E16.5 *(Upregulated)*	Fold Change E18.5 *(Downregulated)*
*Asz1*	2.412	2.537	*Cnmd*	2.715	3.766
*Dnajb11*	1.648	1.682	*Crispld2*	2.418	2.093
*Dnajc3*	1.834	1.582	*Gpx3*	2.748	2.012
*Gm12618*	1.891	1.604	*Spon1*	2.820	2.248
*Gm773*	3.337	2.159	*Porcn*	1.893	2.205
*Noct*	2.221	1.749	*Tatdn2*	2.430	2.165
*Prl2c4*	1.857	1.548	*H6pd*	2.003	2.591
*Prl7a2*	4.394	1.938	*Siglecg*	2.097	2.017
*Prl8a8*	1.710	1.995	*Sphk1*	2.080	2.344
*Rab27a*	1.797	2.143	*1700064E03Rik*	3.457	2.950
*Tmsb10*	2.877	1.703			
*Prom1*	1.579	1.558			

(Fold-Change = Linear Scale; FDR < 0.05)

**Table 3.** Functional Classes of Downregulated Genes (non-inclusive)

**Brain development/function**	*Ntrk2, Elavl4, Gata4, Nog, Nefm, Negr1, Cnrip1, Syt12, Fzd6, Slc26a4, Slc6a15, Amigo2, Hes1*
**Solute transporters**	*Slc26a4, Slc10a6, Slc28a3, Slc2a10, Slc45a3, Slc1a6, Slc22a23, Slc22a4, Slc43a3, Slc1a5, Slc39a4, Slc44a3, Slc7a14, Slc7a6, Slc66a3, Slc27a3, Slc40a1, Slc5a2, Slc6a14, Slc5a6, Slc8a1, Slc20a2, Slc19a2, Slc16a5, Slc7a10*
**Ion channels**	*Trpv6, P2rx4, P2rx1, Orai1, Kcnmb1, Kcnd3, Trpc4, Ano1, Scnn1a, Cacna1g, Kcnmb2*
**GPCR signaling (receptors and mediators)**	*Adora1, F2rl1, Gcgr, Or51e2, Cysltr2, Tbxa2r, Gprc5a, Gprc5b, Gpr146, Mrgprf, Mrgprg, Rxfp1, Adgra2, Adgrl4, Lgr4*
**Plasma Membrane Signal Transduction**	*Wnt2, Tspan1, Tspan2, Tspan4, Tspan6, Tspan7, Tspan12, Pdgfb, Pdgfc, Smo*

**Table 4.** Curated KEGG Terms Associated With Upregulated Genes

KEGG Term	E16.5 Fold Enrichment	E16.5 FDR	E18.5 Fold Enrichment	E18.5 FDR
Ribosome	6.63	3.82E-17	9.28	8.62E-45
Coronavirus Disease-COVID 19	5.07	3.60E-13	6.81	1.44E-35
Glycolysis/Gluconeogenesis	5.82	2.77E-04	---	---
Antigen processing/presentation	5.65	3.28E-05	---	---
Allograft rejection	6.74	3.28E-05	---	---
Graft versus host disease	6.74	3.28E-05	---	---
Biosynthesis of amino acids	4.11	1.55E-02	---	---
Pentose phosphate pathway	5.65	2.50E-02	---	---
Amino and nucleotide sugar metabolism	5.75	1.55E-02	---	---
Phagosome	2.27	2.98E-02	---	---
Cell adhesion molecules	3.09	8.41E-03	---	---
HIF-1 signaling pathway	3.40	2.13E-02	---	---
VEGF signaling pathway	4.42	2.50E-02	---	---

**Table 5.** Canonical Pathways Associated with Cardiovascular and Brain Development

**Cardiovascular Development**	Pathway	E16.5		E18.5	
		P-value (BH)	Z-score	P-value (BH)	Z-score
	Rho GTPase Cycle	0.14	−5.38	0.00068	−7.21
	Myocardin Signaling Pathway	0.18	−2.53	0.00144	−2.68
	Cardiomyocyte Differentiation via BMP Receptors	0.17	−2.24	0.03	−2.65
	Hey1 Signaling Pathway	0.56	−0.33	0.0092	−2.40
	ABRA Signaling Pathway	0.17	−1.67	0.02	−2.14
**Brain Development**	Pathway	P-value (BH)	Z-score	P-value (BH)	Z-score
	Rho GTPase Cycle	0.14	−5.38	0.00068	−7.21
	Axonal Guidance Signaling	0.37	----	0.01	----

(Filtered for B-H-adjusted p-value < 0.05 at one or more gestational ages)

**Table 6.** Upregulation of Fibronectin-associated Genes

Genes	E16.5		E18.5	
	Fold Change *(Linear)*	FDR	Fold Change *(Linear)*	FDR
*Fn1*	---	---	+1.949	0.00143
*Galnt5*	+2.598	0.0160	---	---
*Galnt6*	---	---	+2.582	0.0033
*Galnt12*	+1.696	0.0144	+2.644	0
*Galnt14*	+3.17	0.00003	+3.214	0.00001
*Galnt15*	+2.7	0.0016	---	---
*B4galnt2*	---	---	+4.413	0.0003
*St3gal3*	+2.1	0.0272	+2.465	0.0025
*St6gal1*	---	---	+1.561	0.0186
*Mgat1*	+1.763	0.0070	---	---

**Table 7.** Representative upstream regulators illustrating opposing predicted activation states of vascular and developmental signaling versus inflammatory/stress-associated pathways in *Plac1*-null placentas. Representative IPA-predicted upstream regulators associated with downregulated and upregulated DEG sets in *Plac1*-null placentas.

Downregulated Genes			Upregulated Genes		
Upstream Regulator	E16.5 *(Z-score)*	E18.5 *(Z-score)*	Upstream Regulator	E16.5 *(Z-score)*	E18.5 *(Z-score)*
*Vegf*	−5.76	−6.91	*Il1b*	+4.41	+6.19
*Hgf*	−3.45	−5.30	*Rela*	+2.45	+4.76
*Igf1*	−2.98	−3.82	*Hif1a*	+4.46	+4.90
*Tgfb1*	−2.44	−5.26	*Rictor*	−2.57	−6.03
*Fgf2*	−1.27	−3.25	*Larp1*	−5.57	−8.06
*Bmp4*	−3.78	−3.88	*Lats*	−2.12	−3.05
*Srf/Mrtfb*	−3.49/−3.51	−4.66/−5.43	*Il6*	+3.25	+4.33
*Yap1*	−2.96	−4.55	*Map3k8*	+1.54	+2.77
*Tead2*	−2.24	−4.12	*Ikbke*	+2.63	+2.80
***Tnf***	−2.56	−4.60	***Tnf***	+5.40	+6.16
***Ifng***	−3.01	−5.20	***Ifng***	+4.51	+4.82

Regulators were selected from filtered upstream regulator results with FDR < 0.05 and an absolute IPA activation Z-score ≥ 2 in at least one gestational age.

Z-scores represent IPA-predicted activation states inferred from downstream target-gene expression patterns and should not be interpreted as direct evidence of regulator activation or inhibition.

## Data Availability

The raw and processed microarray data have been deposited in GEO and are openly available (accession: GSE308499). The curated microarray data summarized in this manuscript are included within the article and its Supplementary Materials. The original electron micrographs used in the manuscript are also provided in the Supplementary Materials. An earlier version of this work was posted on bioRxiv in April, 2026. DOI: https://doi.org/10.64898/2026.04.30.721637.
