# Supplementary material for: Transcriptomic Analysis of Plac1 Ablation Reveals Broad Alterations in Signaling Pathways Essential for Prenatal Development and Overlap with a Preeclampsia-Associated Signature
Source: bioRxiv. 2026 Aug 5:2026.04.30.721637. Preprint. [Version 6] doi: 10.64898/2026.04.30.721637 (PMC13232179; doi:10.64898/2026.04.30.721637)
Supplement: Supplementary file 1 [file media-1.zip › Figure_S2_IPA_CompAnalysis_16.5x18.5_UpReg_Canonical_Pathways.pdf]

Figure S2. Heatmap of IPA Canonical Pathways Associated with Upregulated Genes at E16.5 and E18.5

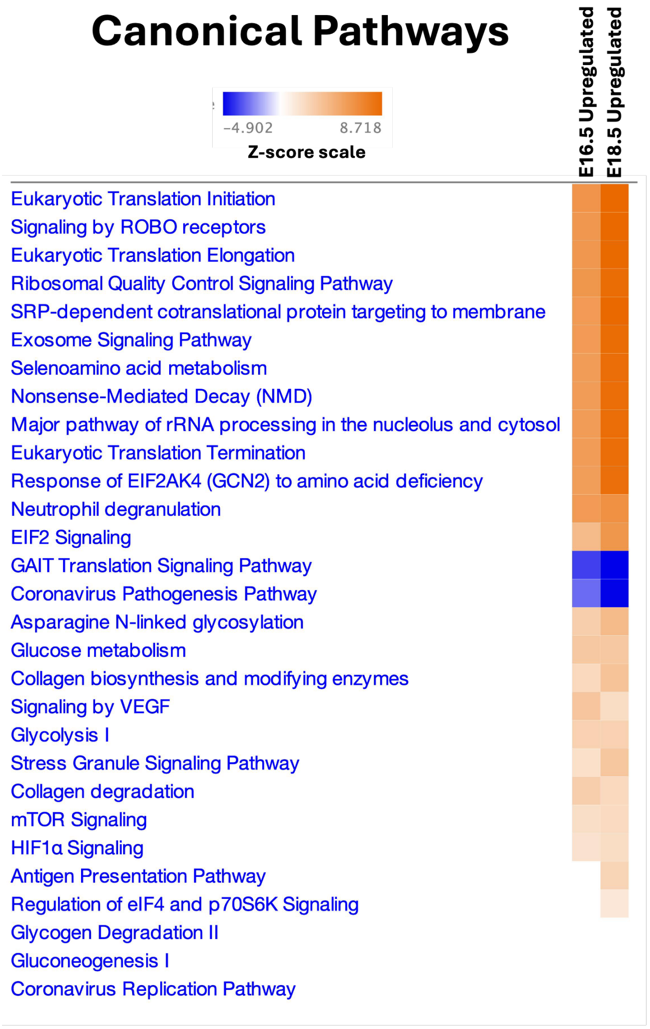

Figure S2. Heatmap of IPA Canonical Pathways Associated with Upregulated Genes at E16.5 and E18.5

Heatmap depicting the IPA “Comparison Analysis” of developmental stage-specific dysregulation of canonical pathways in KO placentas based on upregulated genes.

Color intensity reflects activation Z-scores: blue indicates negative Z-scores (predicted inhibition), and orange indicates positive Z-scores (predicted activation).

Pathways filtered for absolute Z-scores > 2 and Benjamini–Hochberg adjusted p-values < 0.05 at one or more developmental stages and ranked by absolute Z-score.
