# Supplementary material for: Plac1 Ablation Disrupts Signaling Pathways Essential for Prenatal Development and Induces a Preeclampsia-associated Transcriptomic Signature
Source: bioRxiv. 2026 May 28:2026.04.30.721637. Preprint. [Version 3] doi: 10.64898/2026.04.30.721637 (PMC13232179; doi:10.64898/2026.04.30.721637)

Figure S1. Real Time – qPCR: Single gene plots with native scales

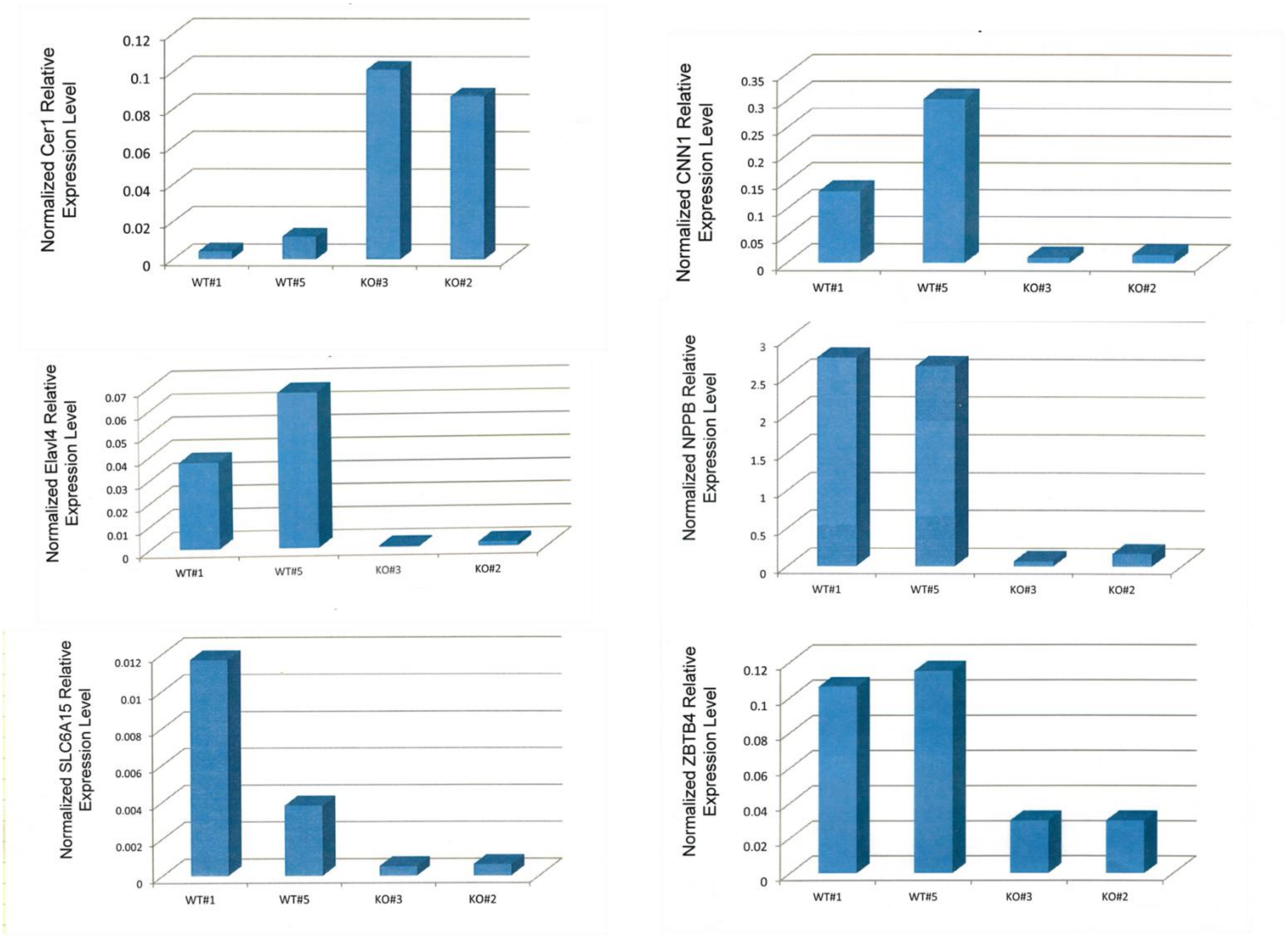

Supplement: Supplementary file 1 [file media-1.zip › Archive/Figure_S1_qPCR Validation for Microarray Results.pdf]
