# Supplementary material for: Plac1 Ablation Disrupts Signaling Pathways Essential for Prenatal Development and Induces a Preeclampsia-associated Transcriptomic Signature
Source: bioRxiv. 2026 May 28:2026.04.30.721637. Preprint. [Version 3] doi: 10.64898/2026.04.30.721637 (PMC13232179; doi:10.64898/2026.04.30.721637)
Supplement: Supplementary file 1 [file media-1.zip › Archive/Jackman_etal_Plac1_v2.1.zip!/00_README_v2.pdf]

README – Supplementary Materials

This directory contains Supplementary Tables and Supplementary Figures associated with the manuscript *“Plac1* Ablation Disrupts Signaling Pathways Essential for Prenatal Development and Induces a Preeclampsia-associated Transcriptomic Signature”

Supplementary Tables include processed expression matrices **(Tables S1-S3)**, differential gene expression results **(Tables S4-S7)**, and GO **(Tables S8-S13)**, KEGG **(Tables S14-S18)**, and Ingenuity Pathway Analysis (IPA) outputs **(S19-S29)** referenced in the main text.

Supplementary Figures include RT–qPCR validation data **(Figure S1)**, IPA comparison visualizations **(Figure S2)**, original electron micrographs corresponding to Figure 10 in the manuscript (**Figures S3-S8)**, and images depicting cardiomegaly in an adult *Plac1*-null male **(Figure S9)**.

File names and indexing of all Supplementary Tables and Figures are provided below and in the Supplementary Materials section of the manuscript.

File List:

Table_S1_Best_oligo_expression_matrix.txt

Table_S2-Anova_Differential_Expression_Plac1_KO.txt

Table_S3_PCA_output_Plac1_KO.txt

Table_S4_Genes_downreg_in_KOvsWT_malesE16.5_1.5fold_v3-1.txt

Table_S5_Genes_upreg_in_KOvsWT_malesE16.5_1.5fold_v3-1.txt

Table_S6_Genes_downreg_in_KOvsWT_malesE18.5_1.5fold_v3-1.txt

Table_S7_Genes_upreg_in_KOvsWT_malesE18.5_1.5fold_v3-1.txt

Table_S8_E16.5_GO_DownReg_Terms_Stats_Genes.tsv

Table_S9_E16.5_GO_UpReg_Terms_Stats_Genes.tsv

Table_S10_E16.5-GO_DysReg_Terms_Stats_Genes.tsv

Table_S11_E18.5_GO_DownReg_Terms_Stats_Genes.tsv

Table_S12_E18.5_GO_UpReg_Terms_Stats_Genes.tsv

Table_S13_E18.5_GO_DysReg_Terms_Stats_Genes.tsv

Table_S14_E16.5_KEGG_UpReg_Pathways_Stats_Genes.tsv

Table_S15_E16.5_KEGG_DysReg_Pathways_Stats_Genes.tsv

Table_S16_E18.5_KEGG_DownReg_Pathways_Stats_Genes.tsv

Table_S17_E18.5_KEGG_UpReg_Pathways_Stats_Genes.tsv

Table_S18_E18.5_KEGG_DysReg_Pathways_Stats_Genes.tsv

Table_S19_IPA_E16.5_DownReg_Canonical_Pathways.xls

Table_S20_IPA_E16.5_UpReg_Canonical_Pathways.xls

Table_S21_IPA_E18.5_DownReg_Canonical_Pathways.xls

Table_S22_IPA_E18.5_UpReg_Canonical_Pathways.xls

Table_S23_IPA_CompAnalysis_16.5x18.5_UpReg_Canonical_Pathways.xls

Table_S24_IPA_Summary_E16.5_DownReg.xls

Table_S25_IPA_Summary_E18.5_DownReg.xls

Table_S26_IPA_E16.5_Downregulated_Upstream Regulators_2026-05-23.csv

Table_S27_IPA_E16.5_Upregulated_Upstream Regulators_2026-05-23-1.csv

Table_S28_IPA_E18.5_Downregulated_Upstream Regulators_2026-05-23-2.csv

Table_S29_IPA_E18.5_Upregulated_Upstream Regulators_2026-05-23-3.csv

Figure_S1. qPCR Validation of Microarray Results.pdf

Figure_S2_IPA_CompAnalysis_16.5x18.5_UpReg_Canonical_Pathways.jpeg

Figure_S3_EM_Fig_10A_WT-PLB94-2b-4-6Kx.jpeg

Figure_S4_EM_Fig_10B_WT-PLB94-2b-5-15Kx.jpeg

Figure_S5_EM_Fig_10C_WT_50Kx-PLB94-2b-5-50Kx.jpeg

Figure_S6_EM_Fig_10D_KO_PLB94-7a maternal fetal interface-1 10k x.jpeg

Figure_S7-EM_Fig_10E_KO_PLB94-7a maternal fetal interface-1 30k x.jpeg

Figure_S8_EM_Fig_10F_KO_PLB94-7a maternal fetal interface-4-25k x.jpeg

Figure_S9_Cardiomegaly_Adult_KOxWT.pdf
